# Effect of intracellular loop 3 on intrinsic dynamics of human β_2_-adrenergic receptor

**DOI:** 10.1186/1472-6807-13-29

**Published:** 2013-11-09

**Authors:** Ozer Ozcan, Arzu Uyar, Pemra Doruker, Ebru Demet Akten

**Affiliations:** 1 Computational Science and Engineering Program and Polymer Research Center, Bogazici University, Istanbul, Turkey; 2 Department of Chemical Engineering and Polymer Research Center, Bogazici University, Istanbul, Turkey; 3 Department of Bioinformatics and Genetics, Faculty of Natural Sciences and Engineering, Kadir Has University, Cibali 34083, Istanbul, Turkey

**Keywords:** ICL3, Molecular dynamics simulation, Transmembrane helix 6, G-protein binding site, Ligand docking, Essential dynamics

## Abstract

**Background:**

To understand the effect of the long intracellular loop 3 (ICL3) on the intrinsic dynamics of human β_2_-adrenergic receptor, molecular dynamics (MD) simulations were performed on two different models, both of which were based on the inactive crystal structure in complex with carazolol (after removal of carazolol and T4-lysozyme). In the so-called *loop* model, the ICL3 region that is missing in available crystal structures was modeled as an unstructured loop of 32-residues length, whereas in the *clipped* model, the two open ends were covalently bonded to each other. The latter model without ICL3 was taken as a reference, which has also been commonly used in recent computational studies. Each model was embedded into POPC bilayer membrane with explicit water and subjected to a 1 μs molecular dynamics (MD) simulation at 310 K.

**Results:**

After around 600 ns, the *loop* model started a transition to a “very inactive” conformation, which is characterized by a further movement of the intracellular half of transmembrane helix 6 (TM6) towards the receptor core, and a close packing of ICL3 underneath the membrane completely blocking the G-protein’s binding site. Concurrently, the binding site at the extracellular part of the receptor expanded slightly with the Ser207-Asp113 distance increasing to 18 Å from 11 Å, which was further elaborated by docking studies.

**Conclusions:**

The essential dynamics analysis indicated a strong coupling between the extracellular and intracellular parts of the intact receptor, implicating a functional relevance for allosteric regulation. In contrast, no such transition to the “very inactive” state, nor any structural correlation, was observed in the *clipped* model without ICL3. Furthermore, elastic network analysis using different conformers for the *loop* model indicated a consistent picture on the specific ICL3 conformational change being driven by global modes.

## Background

As the largest family of membrane proteins in the human genome, the G protein coupled receptors (GPCRs) are structurally characterized by the presence of seven membrane-spanning α-helical segments with an extracellular N terminus and an intracellular C terminus. Upon binding to agonists, a series of conformational changes propagate along transmembrane helices and reach the intracellular part of the receptor, which directly interacts with the hetero-trimeric G-protein. Consequently, G protein’s activation triggers different cascades of events depending on the type of agonists bound to the receptor. Therefore, as the initiation point to the flow of signals into cells, GPCRs are associated with a plenty of diseases that make members of this family significant pharmacological targets.

The first solved X-ray crystal structure of GPCR belongs to bovine rhodopsin [1,2], which is followed by the crystal structure of human β_2_-adrenergic receptor (β_2_AR) in the inactive state [3,4]. Since 2007, the cholesterol bound form of β_2_AR (PDB:3D4S) [5], the structure of turkey β_1_-adrenergic receptor (PDB:2VT4) [6], the structure of a methylated β_2_AR (PDB:3KJ6) [7] and various forms of inactive states of β_2_AR bound to antagonists such as ICI 118,551 and alprenolol (PDB:3NY8,3NY9,3NYA,3PDS) [8,9] have been reported. Finally, the nanobody-stabilized active state of β_2_AR in complex with G-protein, has been solved by Rasmussen and his coworkers (PDB:3SN6) [10,11]. Still, these static pictures of the receptor remain insufficient to describe the dynamic character of the receptor, which governs the function. It is a well-established concept that proteins have an intrinsic ability to sample an ensemble of distinct conformations in order to perform certain functions [12]. The ligand simply selects the optimal receptor conformation for binding followed by an induced fit to stabilize the final conformation. Many questions remain on these multiple, ligand-specific conformational states of β_2_AR with different levels of activity from fully active to fully inactive, which induce distinct signaling pathways.

The ternary complex model proposed in 1980 by Lefkowitz and his coworkers [13] describes an allosteric mechanism for receptor activation. The agonist molecule, when bound to the extracellular part, simply promotes and stabilizes the high affinity β_2_AR-G protein complex. Following the laws of thermodynamics, binding of G-protein increases the receptor’s affinity for agonist binding to the same extent. Fluorescence spectroscopic studies of β_2_AR by Ghanouni *et al.*[14] presented a model with multiple, agonist-specific receptor states, in which the activation occurs through a sequence of conformational changes. They also suggested that the activation barrier for transition from intermediate to active state is high, and that *in vivo* the barrier is more likely reduced by G protein binding. The presence of an intermediate state is further supported by the fluorescence spectroscopy studies of Swaminath *et al.*[15,16], suggesting a mechanistic model for GPCR activation, where agonist binding stabilizes a series of conformational states with distinct cellular functions.

In addition to experiments, several MD simulation studies have been conducted after the inactive and active states of the receptor have been solved by X-ray crystallography. One simulation study by Dror *et al.*[17] reveals that the receptor exists between two distinct inactive conformations of the receptor, one with the ionic lock intact and one with the lock broken. In 2011, Dror and his coworkers proposed a completely different activation mechanism in which the structural changes start at the G protein binding site propagating upwards as opposed to agonist-induced conformational changes that start at the agonist binding site and propagate down to G protein binding site [18]. The agonist-bound crystal structure of β_2_AR without a binding partner (PDB:3PDS) recently revealed by Rosenbaum *et al*.[9] is found to be identical to the inactive state of the receptor (PDB:2RH1). This suggests that in the absence of a G-protein, the receptor prefers to adopt the inactive conformation whether or not it is bound to an agonist. In other words, the agonist molecule is not sufficient alone to shift the equilibrium to the active state. Dror *et al.*[18] also proposed an intermediate state for G-protein binding site, which exists as a part of the receptor’s intrinsic dynamics. Binding of a G-protein to this binding site simply promotes a transition to the active conformation, which is further stabilized by an agonist bound at the extracellular region. The most important feature about the dynamics of β_2_AR is the strong coupling that exists between the intracellular G-protein binding site and the extracellular ligand-binding site of the receptor [7,19]. The receptor behaves like a pair of pincers where the intracellular part becomes narrower as the extracellular part becomes wider, and vice versa.

Due to its unstructured nature, ICL3 region is either unresolved in crystallographic experiments or completely removed and replaced by T4-lysozyme (T4L) to facilitate the crystallization. Thus, none of the experimental and simulation studies have discussed the possible effect of ICL3 on the intrinsic dynamics of the receptor. Its replacement by T4L to facilitate crystallization did not prevent agonist-induced conformational changes based on fluorescence spectroscopy measurements [20]. Yet, it is well accepted that its direct interaction with G-protein probably have a significant role on the receptor’s dynamics and the activation/inactivation pathway [21,22].

In this study, the effect of ICL3 on receptor’s conformational dynamics was investigated via two distinct models of the receptor. Both models were generated from the inactive state of the receptor (PDB:2RH1) after removal of T4L. Moreover, the partial inverse-agonist carazolol was removed from the binding site of both models, since the goal of this work was to provide data about the intrinsic dynamics of the receptor, i.e., the ensemble of conformations accessible to its apo form. According to the current view on ligand binding, the equilibrium distribution of conformational states may be shifted upon ligand binding.

In the so-called loop model, the ICL3 region was modeled as an unstructured loop of 32-residues length and inserted between two open ends of TM5 and TM6 (residues 230 and 263), whereas in the second model, these two open ends were “clipped” or simply covalently attached to each other. The “clipped” model of the receptor, serving as a reference in our study, has been commonly used in recent simulation studies as well [18,23]. Both models were subjected to 1 μs MD simulation in a POPC membrane bilayer at 310 K. The essential dynamics analysis was carried out to reveal important allosteric coupling within the receptor in the presence of ICL3. Two distinct snapshots taken from the loop model’s trajectory were further used as docking targets for an agonist and an antagonist molecule in order to investigate the effect of ICL3 on binding site conformations. Finally, elastic network analysis was performed on different conformations and loop models to reveal a consistent picture on receptor intrinsic dynamics.

## Results and discussion

### Presence of ICL3 affects RMSDs and loop mobility

**Figure 1:**
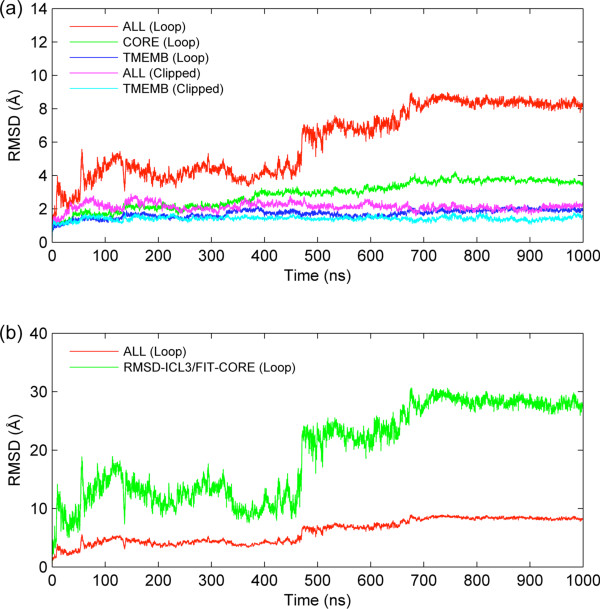
**RMSD profiles for the loop and clipped model simulations. (a)** RMSDs plotted based on alignments using different regions (ALL, CORE: without ICL3, TMEMB: transmembrane helices) of the receptor. **(b)** RMSD of ICL3 only (green line).

The difference between loop and clipped model dynamics is illustrated in the root mean square deviation (RMSD) profiles throughout the simulation. In Figure 1a, three different RMSD values are plotted for the loop model after fitting all snapshots to the initial snapshot based on coordinates of either the whole protein (red line: ALL Loop), the core region excluding ICL3 (green line: CORE Loop) or the transmembrane region composed of helices (blue line: TMEMB Loop). The large RMSDs observed in loop model (ALL) are due to the presence of ICL3. The core region of the loop model is equivalent to the clipped model and thus, the comparison between their RMSD profiles (CORE Loop and ALL Clipped) reveals that the clipped model has reached a plateau at an earlier time (~200 ns) than the loop model (~700 ns). The time at which the loop model’s core region reaches the plateau corresponds to the time when ICL3 stabilizes as well. The stabilization of ICL3 corresponds to a significant change in its conformation, observed as close packing underneath the receptor, which will be discussed later in the text.

**Figure 2:**
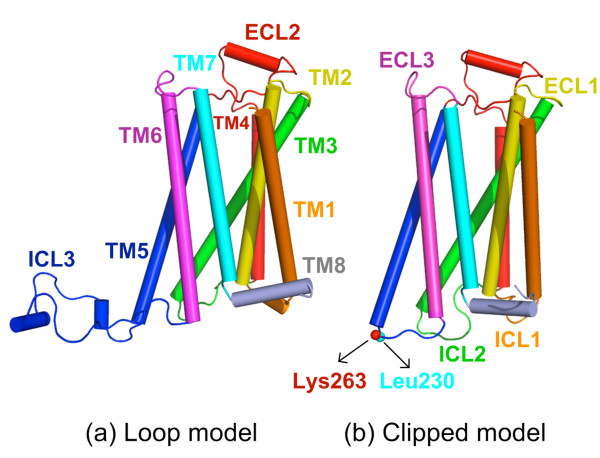
**Two β**_**2**_**AR models used in MD simulations. (a)** Loop model with the missing ICL3 modelled using MODELLER, **(b)** Clipped model without ICL3.

On the other hand, the RMSD values of the transmembrane region (TMEMB) reach a plateau at around 50 ns in both models. This indicates that the structure of transmembrane region is preserved comparably in both models. Furthermore, the presence of ICL3 affects the mobility of small intra- and extracellular loops (ICL1, ICL2, ECL1, ECL2 and ECL3, see Figure 2 described in Methods) in the loop model, which are present in the CORE profile but not the TMEMB. There is a strong correlation between the RMSD profiles of the whole protein and ICL3 (only), given by the uppermost red and green lines in panels A and B, respectively. The RMSD value of ICL3 is obtained after alignment of the core region to the initial structure (RMSD-ICL3/FIT-CORE; green line in Figure 1b). Thus, the extremely high RMSD of whole receptor with ICL3 is a consequence the high mobility of the long intracellular loop.

**Figure 3:**
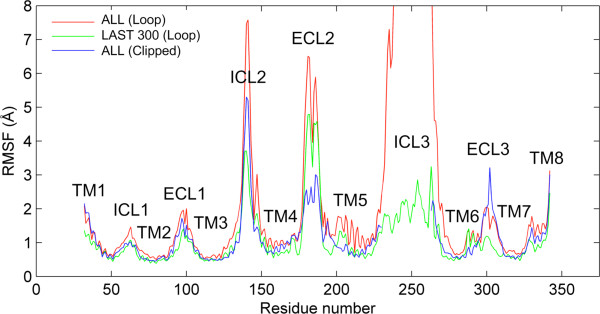
**RMSF profiles of residues in the loop and clipped models.** Fluctuations are calculated using the whole 1 μs trajectory (ALL) or the time frames of 0.7-1 μs (LAST 300). Presence of ICL3 imparts mobility to other loops of the receptor. Packing of ICL3 under the receptor hinders fluctuations after 700 ns.

The root mean square fluctuation profiles (RMSF) are plotted using the time range as [50 ns-1000 ns] for both models (Figure 3). The RMSF of each alpha-carbon atom in the protein is calculated based on the average structure of the aligned snapshots. The first 50 ns are excluded as the equilibration stage of the transmembrane region in both models (see Figure 1a). The extent of the average fluctuation during 950 ns is found to be higher in the loop model (red line) than that of the clipped model (blue line), in almost all protein regions, except the ECL3 region. Another time range for the loop model was taken as [700–1000 ns] (green line; LAST 300), where the whole protein has reached a plateau (see Figure 1b).

The most significant difference between two time ranges for the loop model is in the mobility of the ICL3 region, which fell down to 2–3 Å in the second time range from 16 Å (out of the range of Figure 3). In addition, a relatively lower decrease in RMSF is observed in all parts of the protein including the loops and the more stable helices in the second time range. However, such a difference cannot be observed in the mobility of the clipped model based on the two time frames (not shown). These results indicate that the fluctuation of ICL3 region in the loop model is directly reflected on every part of the protein structure, including the transmembrane regions. Once ICL3 becomes closely packed under the receptor at around 700 ns (see next section), the mobility of the transmembrane region decreases slightly and becomes more similar to that of the clipped model.

Another important observation about the RMSF profiles in Figure 3 is the mobility of the ECL2 loop region, which is at the extracellular side of the membrane and plays an important role as an access point to the binding site. For the loop model, during the last most stable 300 ns, the RMSF of ECL2 decreases to 5 Å from 6.5 Å, as a consequence of the decrease in the mobility of ICL3. But still, the mobility of ECL2 in the loop model is higher than that in the clipped model irrespective of the time ranges considered. The higher ECL2 mobility allows a wider range of conformational sampling, which would include the open/closed forms of the gateway to the binding site, making the loop model’s binding site more accessible and accommodating for diffusing ligands than the clipped model.

### The conformational change of ICL3 gives rise to a “very inactive” state of the receptor

**Figure 4:**
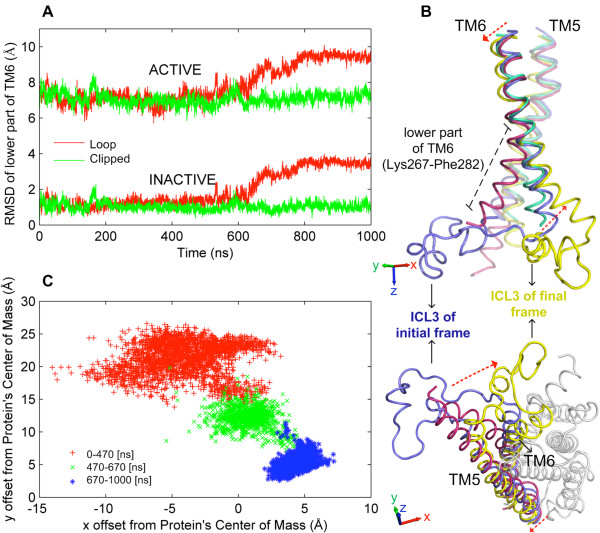
**Conformational change in the intracellular part of TM6. (A)** RMSD profile of the TM6 intracellular part from its inactive and active states in the loop (red) and clipped (green) models. **(B)** Initial (blue) and final (yellow) snapshots from 1 μs loop trajectory, showing TM5 and TM6 in comparison with the inactive (green) and active (magenta) crystal structures. **(C)** Changes in the x-offset with respect to the y-offset representing the change between center of masses of ICL3 and protein’s core.

Figure 4A shows the RMSD profiles of the sixth transmembrane helix (TM6) from its inactive (PDB:2RH1) and its active states (PDB:3SN6) in reported crystal structures. The RMSD is calculated for the intracellular part of the helix composed of residues 267–282. In the loop model (red), the deviation from both inactive and active states starts to increase at around 600 ns and levels off around 800 ns, amounting to a change of 2 Å. On the other hand, no significant change is observed in the clipped model (green). The deviation is illustrated in Figure 4B, where the first snapshot of TM6 (blue) is close to the inactive state (green) and the last snapshot of TM6 (yellow) is found to be away from both the inactive and the wide-open active state (magenta). The second view of the receptor from the intracellular side in Figure 4B shows that ICL3 becomes more wrapped up under the core of the receptor (final frame shown), which will be named as a “very inactive” state inaccessible to G-protein binding.

This close packing of ICL3 is quantitatively represented in Figure 4C, which shows the sudden change in the *x*-offset with respect to the *y*-offset. The value of *x*-offset is the difference in the *x* coordinates between the center of masses for the core of the receptor and the ICL3 region. Similarly, the value of *y*-offset is calculated from the difference in *y* coordinates of these center of masses. Three distinct clusters are observed in time ranges of [0–470] ns, [470–670] ns and [670–1000] ns. The first (red) and third clusters (blue) correspond to the open and packed states of ICL3, respectively. The second one (green) represents a transition between the two states. Interestingly, the second cluster’s starting time at 470 ns corresponds approximately to the time at which small helical formations appear inside ICL3 (see Additional file 1: Figure S1). It is an open question whether these helical formations might trigger the transition to the packed state. Additionally, the onset of the third cluster around 670 ns corresponds to the time, at which TM6 starts to deviate from the reference crystal structures (see Figure 4A).

**Figure 5:**
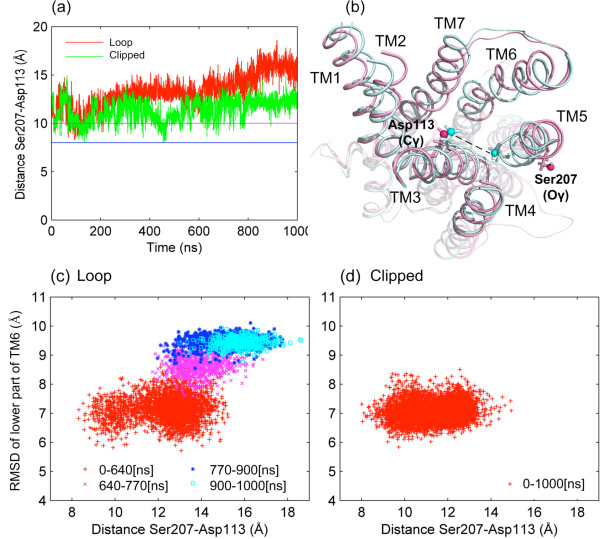
**Conformational change in the binding site. (a)** Ser207(Oγ)-Asp113(Cγ) distance profiles for the loop (red) and clipped (green) models. Blue and purple lines bracket the experimentally determined critical range in active states [25]. **(b)** Conformations representing the open and closed forms of the binding site in loop model. **(c, d)** The correlations between TM6 shift and Ser207-Asp113 distance for the loop and clipped models, respectively.

The changes observed at the intracellular part of the receptor seem to affect the extracellular part, specifically the binding site of the receptor. Figure 5a illustrates the profile of the distance between the pair of residues, Ser207-OG on TM5 and Asp113-CG on TM3. Recent simulation studies by Katritch *et al.*[24] have revealed that tilting of TM5 towards the receptor axis enables an optimum interaction between agonists and the two anchor sites, Asp113/Asn312 and Ser203/Ser204/Ser207 side chains. Based on experimental studies [25], the distance between the side chain oxygen of Ser207 and gamma carbon of Asp113 should be within a range of 8 Å (blue, horizontal line) and 10 Å (purple line) in order to accommodate the agonists at the binding site. However, the distance profile in Figure 5a is most often out of this critical range and even beyond the distance values of the inactive state, which is around 11 Å. At around 600 ns, when the sudden conformational changes in both TM6 and ICL3 occur, this distance starts to increase from 13 Å to 16 Å in loop model (red). On the other hand, there is no significant change in the distance profile of the clipped model (green) after 600 ns as expected.

The variation in the distance between Ser207 and Asp113 is illustrated in Figure 5b (top view, looking down from the extracellular side), which shows two conformations of the receptor, with minimum and maximum distance values of 8.3 Å and 18.6 Å (shown as light blue and purple, respectively). Clearly, the intracellular part of TM5 is slightly moving into the core region (see Figure 5b), while its extracellular part is moving away from the core region of the receptor. As a result, Ser207, which is located at the extracellular part of TM5, drifts away from Asp113 on TM3, position of which does not change notably. Similar motions are observed for TM4 and TM6 as well. As a result, the enlarged binding site becomes unfavorable for agonist binding due to lack of some key interactions.

Figure 5c illustrates the correlation between the TM6 shift at the intracellular part of the receptor and the Ser207-Asp113 distance at the extracellular part of the receptor. In the loop model, the change in the RMSD value of TM6 with respect to the active state happens at around the same time as the increase in the Ser207-Asp113 distance, whereas in the clipped model, no such correlation is observed (see Figure 5d). Three different states of the structure are observed in the loop model at around [0–640], [640–770] and [770–1000] ns intervals. In contrast, the conformational variations of the clipped model remain in a restricted area, which corresponds to the first conformational state ([0–660] ns) of the loop model.

**Figure 6:**
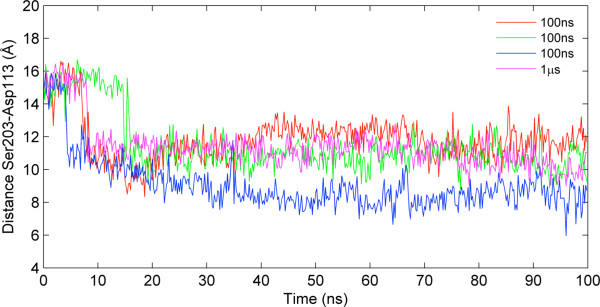
**Ser203(Oγ)-Asp113(Cγ) distance profiles.** The initial distance indicative of a relatively open binding site decreases in all simulations (1 μs and three 100 ns independent trajectories) resulting from the positioning of TM6 and ICL3.

In addition to the 1 μs MD simulation, three independent 100 ns MD simulations with different initial conformations and velocities were performed as explained in Methods. Due to restrained conditions during the preparation stage prior to MD runs, the Ser203-Asp113 distance value was extended to ~16 Å from the initial value of ~11-12 Å. Ser203 is another key residue for binding that lies on the next turn above Ser207 on TM5. The change in the distance between Ser203 and Asp113 is mainly the result of a change in the position of Ser203 (both backbone displacement and side chain rotation). Within the first 20 ns of all four MD simulations (including the 1μs simulation), the distance rapidly decreases back to its initial value of ~11-12 Å as shown in Figure 6. The explanation for such a decrease is that all four simulations started with an expanded extracellular (binding site region) part and an intracellular part with ICL3 set aside. However, the receptor is found to be in equilibrium when its extracellular part is open (wide) and its intracellular part is closed (narrow) with ICL3 closely packed underneath or vice versa. This is the direct consequence of the strong allosteric coupling that exists between extracellular and intracellular regions of the receptor and this seems to be consistent with the ternary complex model suggested by de Lean *et al.*[12].

The loop model shows a conformational variation in its second intracellular loop (ICL2), which is correlated with the motion of ICL3. Additional file 2: Figure S2 shows four different stages of conformational variation of ICL2 changes with respect to intracellular part of TM6. Both RMSD values are calculated with reference to the active state (PDB:3SN6). However, no such major structural change is observed in the clipped model. The motion of ICL2 in the loop model is also illustrated, in which the ICL2 between TM3 and TM4 steps aside as the ICL3 comes closer to the middle region (initial stage: blue, second stage (at 700 ns): cyan, final stage: red, and active crystal: green). No such conformational rearrangement in ICL2 is observed in the clipped model. Additional file 3: Figure S3 illustrates the change in the RMSD value of ECL2 with the change in the Ser207-Asp113 distance. As the distance increases in the loop model, there is a conformational variation in ECL2 with respect to the active state. However, no such correlation is observed in the clipped model. Clearly, the structural variation in ECL2 is directly affected by the change in the distance as a result of a shift of TM5 away from the binding site, which is in turn a consequence of the ICL3 motion and TM6 shift at the intracellular part of the receptor.

### Ionic lock (Arg131-Glu268) is not a molecular switch

During 1 μs long MD simulation of the loop and clipped models, which represent the inactive state of the receptor, the ionic lock profiles are monitored as shown in Additional file 4: Figure S4. Consistent with previous work [18], the ionic lock seems to be on and off during the simulations of both models (in upper panels). Thus, this ionic lock cannot distinguish between active and inactive states. The cause behind the breakage/formation of this ionic lock is found to be the result of a change in the rotational state of the *Χ* angle of Glu268, which coordinates perfectly well with the ionic distance profiles (in lower panels).

Furthermore, the increase in the distance between two side chains that form the ionic lock, namely Arg131-N and Glu268-O, coincides properly with the increase in the distance between their alpha-carbons. In the profiles of the inactive state, the backbone distance fluctuates at around 9.5 Å and reaches 12.4 Å at most. However, in the known crystal structure of the active receptor (PDB:3SN6), the distance between alpha-carbons is around 16 Å as a result of a significant outward shift in the intracellular part of TM6. Thus this backbone distance could be one possible measurement for detection of activation.

### Essential dynamics analysis reveals the transition to the “very inactive” state in the first principal mode

**Figure 7:**
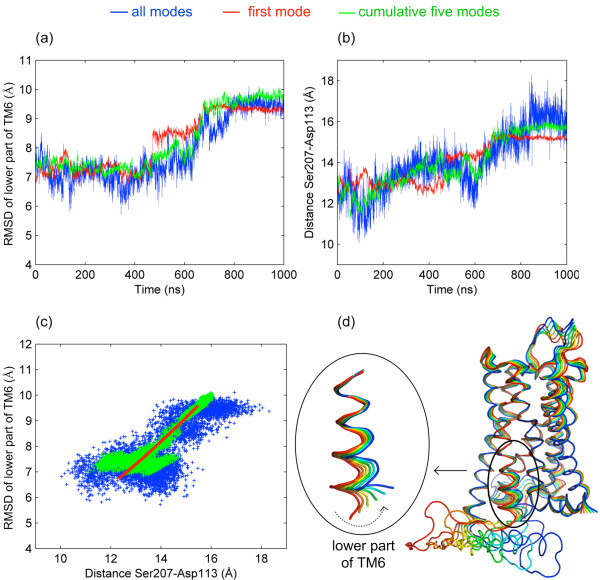
**Essential dynamics of the loop model. (a)** RMSD profiles for the intracellular part of TM6 in the loop model’s original trajectory and after the projection onto the first and the cumulative five principal modes. **(b)** Original and reconstructed profiles for the distance between Ser207(Cα) and Asp113(Cα). **(c)** The correlation plot between RMSD of TM6 and Ser207-Asp113 distance. **(d)** Projection of the loop model’s trajectory onto the first principal mode, shown as harmonic motion.

For both models, each frame in the trajectory was aligned onto the initial structure. Then principal component analysis (PCA) based on only Cα atom coordinates was performed to understand the effect of ICL3 on the essential dynamics of the receptor [26]. The first principal mode explains about 69% and 22% of the protein’s overall motion in the loop and clipped models, respectively. Figure 7a displays three different RMSD profiles for the intracellular part of TM6 in the loop model after alignment on the active state. These are computed for the original trajectory (blue, same profile as in Figure 4A) and for the two reconstructed trajectories, one including only the first mode (red) and the other showing the cumulative effect of the first five modes (green). Projection of the MD trajectory onto the first principal eigenvector (red) shows an abrupt change, which is coupled with the transition to the “very inactive” conformation. The profile obtained from the projection of cumulative five modes, as expected, explains this transition better.

Additionally, the distance profiles between Ser207 and Asp113 in the loop model was recalculated using Cα atoms only as shown in Figure 7b. A high correspondence between the original and the two reconstructed profiles is observed as in Figure 7a. Also, a plot of RMSD value versus the Ser207-Asp113 distance clearly shows that the essential modes (first and cumulative five) describe the distribution in the original trajectory (Figure 7c). Thus, the closure of the ICL3 driven by the first mode (Figure 7d) is strongly coupled with the opening of the binding site indicated by the Ser207-Asp113 distance. For the clipped model, same profiles are plotted in Additional file 5: Figure S5. The profiles obtained from the projection of the first and cumulative five modes do not explain satisfactorily the dynamics of the extracellular and intracellular regions of the receptor.

### Elastic network modeling reveals coupling between global modes and ICL3 conformational transition

MD simulations were performed on a receptor model including a specific unstructured conformation of ICL3 obtained from MODELLER. In order to show the independence of the observed phenomena from the initial MD structure, ANM was performed on four distinct conformations of the loop model. These were selected as the initial, average and final structures of the 1 μs-long loop trajectory, and a receptor model containing an alternative unstructured conformation of ICL3, also provided by MODELLER. The RMSD between the alternative loop model and the one used in our MD simulations is ~ 20 Å for the ICL3 residues after an alignment of transmembrane regions.

The correlation between PCA and ANM modes is routinely assessed by the average overlap value

(1) $$O_{\mathrm{ave}}={\left(\frac{1}{k}\sum_{i=1}^{k}\sum_{j=1}^{k}(p_{i}.u_{j}){}^{2}\right)}^{1/2}$$

where **p**_*i*_ and **u**_*j*_ represent the *i*^th^ and *j*^th^ normalized eigenvectors from PCA and ANM, respectively. The squared inner dot products are generally summed over the first *k =10* modes, which describe the collective subspaces of each method. The average overlap values are 0.64, 0.72, 0.62 and 0.66 between the first 10 modes of PCA and ANM performed with the initial, average and final structures of MD run and the alternative loop model, respectively. These values are quite high (relatively closer to 1), representing satisfactory overlap between ANM and PCA subspaces.

**Figure 8:**
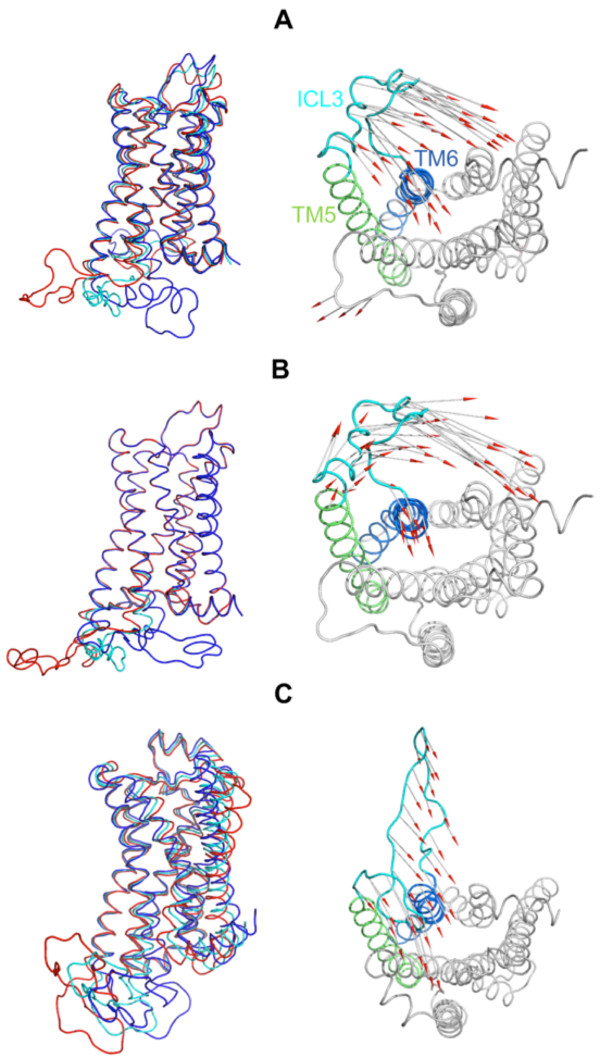
**Collective modes driving ICL3 and TM6 towards the core of the receptor. (A)** The first anharmonic principal mode for the 1 μs MD trajectory (same red and blue conformers as in 7D, cyan representing the average MD structure), **(B)** the second slowest mode from ANM performed on the average structure, and **(C)** the first mode from ANM performed on the alternative loop model. On the left panels, alternative conformations (red and dark blue) are provided together with the average structure (cyan) for the specific mode. The right panels provide vector representations of the same deformations based on average structures (intracellular view).

In Figure 8, the conformational changes of ICL3 and TM6 are shown for specific ANM modes that yield a high overlap value (*O*_1,*j*_ = |**p**_1_. **u**_*j*_|) with the first principal component of MD run. The left and right panels on the figure indicate different views (side and intracellular) for each mode. Specifically, vector representations of deformations, shown from the intracellular part (right panels), indicate clearly the inward movement of ICL3 and TM6 in the 1st mode of MD (Figure 8A), ANM 2nd mode for the average structure (*O*_1,2_ = 0.81, Figure 8B) and ANM 1st mode for the alternative loop model (*O*_1,1_ = 0.57, Figure 8C).

The overlap matrices calculated based on residue displacements of ICL3 and TM6 region only (see Additional file 6: Figure S6) indicate several ANM modes exhibiting high overlap with the first mode of MD. Thus, slow modes of ANM clearly drive the significant conformational change of ICL3 and TM6 towards the receptor core, independent of the ICL3 conformation/model used. In summary, our ANM analysis justifies that the ICL3 dynamics observed in MD run can be attributed to be a feature of intrinsic receptor dynamics in conformity with a recent study carried on catalytic loop motions for different enzymes [27].

### The clustering of MD snapshots reveals more conformational variations in the loop model

**Figure 9:**
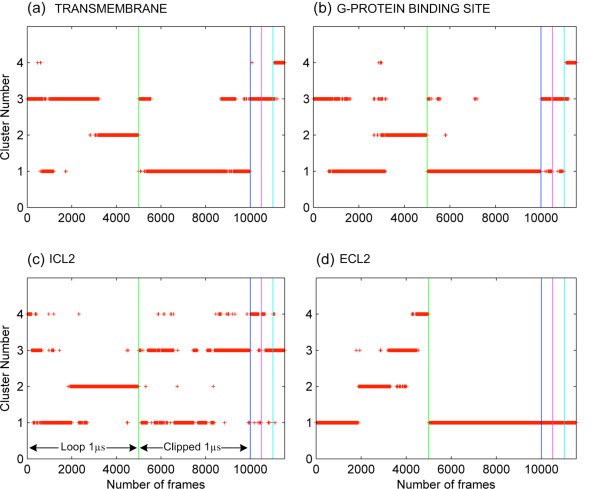
**Clustering profiles of all trajectories based on different regions of the receptor. (a)** transmembrane region, **(b)** G-protein binding site, **(c)** ICL2 and **(d)** ECL2. All graphical representations were prepared using MATLAB [28] in this article.

The snapshots taken from the simulations of the loop and clipped models are clustered all together based on different regions of the receptor: transmembrane region, intracellular part of the receptor, ICL2 and ECL2 loop regions using an RMSD cutoff of 1.8 Å, 1.8 Å, 3.7 Å and 3.3 Å, respectively. The region for the alignment is chosen as the transmembrane region in all four cases. In all cluster profiles shown in Figure 9, the simulation time is divided into five ranges. Frames 1–5000, 5001–10000 and 10001–11500 are taken from the μs-long loop run, μs-long clipped run and the three short runs for the loop model.

The clustering profile of the transmembrane region shows four distinct clusters for 1 μs simulation of the loop model as illustrated in Figure 9a. Two of those clusters dominate over the other two, since they contain 54% and 37% of the total snapshots, which are observed at around [0–600] ns and [600–1000] ns, respectively. On the other hand, two distinct clusters are obtained for the clipped model, and only one of them dominates for 92% of the time. For each of the three short MD simulations, there is only one single cluster that dominates during 100 ns. These results indicate that the transmembrane region of the loop model alternates between two distinct conformations, while the clipped model’s transmembrane region prefers to adopt only one. Interestingly, the second conformation in the loop model observed between 600 and 1000 ns coincides with the time at which the ICL3 changes its conformation and the receptor adopts a “very inactive” state with an expanded binding site. A highly similar clustering profile is obtained for the binding site region as shown in Additional file 7: Figure S7. This is an expected outcome considering that the binding site is embedded in the transmembrane region.

Figure 9b shows the cluster profile of the intracellular part of the receptor, which consists of residues interacting with the G-protein based on the active crystal structure (PDB:3SN6) [29]. Clearly, the loop model’s intracellular part samples three distinct states while the clipped model’s intracellular part only samples one conformation. In the three short simulations, there is also one single conformation dominating the others. The structural flexibility of the intracellular part is critical in making contact with the G protein. For the loop model, three snapshots were selected from each cluster shown in Figure 9b as representatives and illustrated in Additional file 8: Figure S8 with a bottom view to show the contact of the receptor with the helical segment of gamma subunit of G protein. In the active crystal structure (PDB:3SN6) taken as a reference and placed on top of the figure, G protein’s helical segment nicely fits the binding cavity. At the initial stages of the simulation, the binding cavity is almost preserved. Towards the end of the simulation, the motion of ICL3 closes down the G-protein binding site almost completely, as shown in the last frame leaving no contact point for the G protein.

The clustering profile in Figure 9c, shows two dominant clusters for the ICL2 region in both model. The two distinct states in the loop model are sampled for about 29% and 58% of the time, while in the clipped model two major clusters are sampled for 39% and 59% of the time. Each of the short simulations of the loop model still does not show structural variation during 100 ns, similar to other two cases above. These results indicate that ICL3 has no significant effect on the conformational sampling of ICL2.

Finally, the cluster profile in Figure 9d shows four distinct clusters for ECL2 region of the loop model, with each consisting of a considerable amount of snapshots and sampled consecutively during the simulation. On the other hand, the clipped model’s one μs simulation as well as three short simulations of the loop model, impart no conformational variation to the ECL2 region. The ECL2 loop region is the second extracellular loop covering the top of the receptor and plays a critical role of providing a passage to the binding site region. Therefore, the ability of ECL2 to sample various conformations, being a functionally important feature for the receptor, is clearly enhanced in the presence of ICL3.

### Docking results of epinephrine and ICI to an open and a closed form of the binding site

Two frames of the loop model are selected from the 1 μs trajectory to represent the two extreme cases of Ser207-Asp113 distance value (see Figure 5a, b). One of the conformers is a closed form with a distance value of 8.31 Å, which is in the range of active states (8–10 Å) [26], while the other conformer represents an open form with the maximum distance value of 18.63 Å. The docked ligands are a natural agonist epinephrine, and ICI, which is an antagonist with a known crystal conformation (PDB:3NY8). The epinephrine is chosen due to its relatively small size, and ICI is selected because it is a large antagonist with an experimentally determined conformation.

**Figure 10:**
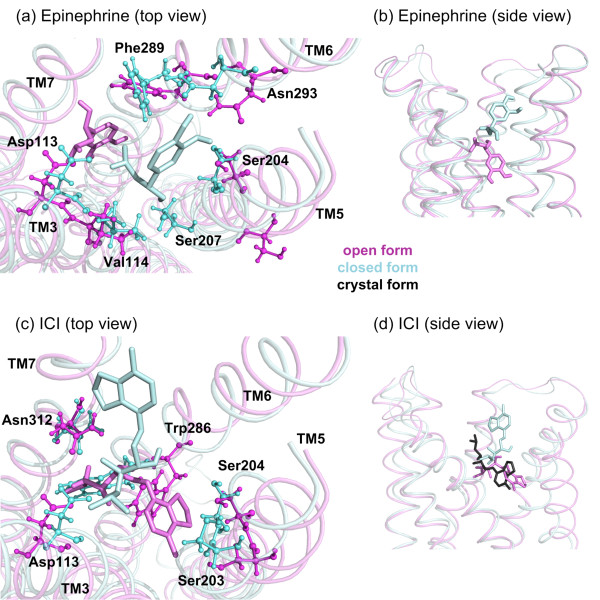
**Best docked poses of ligands. (a, b)** for agonist epinephrine and **(c, d)** for antagonist ICI in open (magenta) and closed (light blue) forms of the receptor, showing both top and side views. Ligands are shown in sticks representation in the same color with its docked structure. Key residues interacting with the ligand are labeled and also shown as ball-and-stick. For comparison, the bound state of ICI in the crystal structure (PDB:3NY8) is shown in black. All molecular graphics were prepared using PyMOL [32] and VMD [33] in this article.

Figure 10a illustrates the poses of epinephrine with highest scores docked to open and closed forms of the receptor (a top view looking down from the extracellular region). Epinephrine is shown as sticks while the key residues are in ball-and-sticks representation. The epinephrine’s highest score conformation docked to closed form (light blue) has more favorable interactions with neighboring residues than the highest score conformation docked to open form (magenta) of the receptor (see Figure 10b for an alternative side view to the structures). There exist a total of eight neighboring residues, which interact with epinephrine in closed form within a radius of 3.5 Å, namely Asp113, Val114, Ala200, Ser204, Ser207, Phe289, Phe290, and Asn293. Seven of them, excluding Ala 200, are known to be key interacting residues in agonist binding [30] (Additional file 9: see Figure S9A, B showing interactions of epinephrine obtained from MOE tool) [31]. On the other hand, the best pose of epinephrine in the open form is found slightly out of the binding site region making interactions with ten residues with a distance of less than 3.5 Å (Asp113, Val117, Phe282, Cys285, Trp286, Phe289, Leu311, Asn312, Gly315, Asn318), among which only three (Asp113, Phe289 and Asn312) are key residues (see Figure 10a). The other seven are not reported as being significant in agonist binding. Considering the small size of epinephrine, the closed form is found to be more favorable than the open form.

The docking results of ICI indicate a complimentary situation. Due to its large size, the antagonist ICI cannot fit into a narrow binding site in the closed form, but it can be favorably docked into a wider binding site, such as the open form. Figure 10C shows the highest score conformations docked in the open (magenta) and closed (light blue) form of the receptor (see Figure 10d for a comparison to the bound state of ICI in the crystal structure shown as black sticks). The RMSD values of docked ICI to open and closed forms with respect to its native state are determined as 3.95 Å and 8.16 Å, respectively. Clearly, ICI when bound into a wider binding site is able to interact with experimentally reported key residues for antagonist binding. ICI interacts with eight residues within a radius of 3.5 Å, Asp113, Tyr199, Ser203, Ser204, Phe208, Trp286, Tyr308 and Asn312, of which five (Asp113, Ser203, Ser204, Trp286, Asn312) are reported as key residues [8] (see Additional file 9: Figure S9 for the specific interactions of ICI). Furthermore, ICI is correctly oriented in the binding pocket with the hydrophobic catechol ring interacting with Ser203 and Ser204, and its polar end interacting with Asp113. However, in the closed form, ICI is improperly positioned in the binding pocket, interacting within a radius of 3.5 Å with Thr110, Asp113, Val114, Phe193, Tyr199, Phe289, Asn293, Lys305 and Tyr308 of which only two (Asp113 and Val114) are known key residues.

To sum up, the agonist is favorably bound to the closed form, which coincides with the ICL3-open conformation during the first half of the simulation. The ICL3-open conformation may correspond to an intermediate state that promotes G-protein binding, which seems to be stabilized by the presence of the agonist at the binding site. An MD simulation, where the binding site would be constrained to the agonist bound geometry, would give more insight in this perspective. In the second half of the simulation, the open geometry of the binding site is observed to which the antagonist favorably docks. This “very inactive” state corresponds to the closely packed ICL3 that completely blocks the G-protein binding site.

## Conclusions

The crystal structure of β_2_AR has been resolved for the first time in 2007, and since then in silico studies have been conducted to unravel structure-dynamics-function relationship of this G-protein coupled receptor. However, the missing intracellular loop ICL3, which is known to interact with the G protein at the cytoplasmic side, has not been considered or elaborated so far in these studies. In this respect, our MD study exposed the marked effect of ICL3 on collective dynamics and justified the correlated motion between the intracellular G-protein binding site and the extracellular ligand-binding site of the receptor [7,19]. The receptor behaves like a pair of pincers where the intracellular part becomes narrower as the extracellular part becomes wider, and vice versa.

In accordance with this coupling behavior, our μs long MD simulation of β_2_AR, which included the modeled intracellular loop ICL3, revealed a so-called “very inactive” state of the receptor, which has not been reported before. In the second half of the simulation, the ICL3 moved toward the core of the receptor and completely blocked the G-protein binding site. Consequently, the intracellular part of TM6, adjacent to ICL3, also shifted toward the core of the receptor. This conformational change in TM6 is in the opposite direction with respect to experimentally observed deformation during activation [9], which is observed as an expansion or outward movement towards the lipid membrane. This new inactive state of the receptor may provide insight into the design of novel therapeutic drugs.

Another important observation is the correlated motion between the binding site and the G-protein binding site regions. At around the same time when the ICL3 blocked the G-protein binding site, the extracellular binding site of the receptor expanded. The expansion was detected based on the distance profile between two anchor residues, Ser207 and Asp113, critical for agonist binding. In line with these findings, our docking studies indicated favorable antagonist binding to the expanded binding site (closed ICL3) and agonist binding to the closed binding site (open ICL3).

This coupled conformational change seems to be transmitted from the intracellular part to the extracellular part of the receptor via TM5 and TM6. As Sara Linse stated in her review [34], “*a 7TM receptor is like a bundle of rods immersed in the membrane and if a ligand grips the bundle at one end, the bundle opens up like a bouquet of roses at the other end”.* In our study, the intracellular end is gripped or held tight by ICL3, and consequently the extracellular part opened up. In other words, ICL3 played the dominant role in inducing the change in the intracellular part, which induced in turn the extracellular part. This dominancy of ICL3 is expected due to its high mobility, which is also a desirable quality for initiation of interactions with intracellular proteins [20,21]. Thus, we suggest that when left without ICL3, the receptor would not be able to sample that *inactive state* at all.

This transition to the “very inactive” state took place within a time frame of about 0.1 μs (starting at ~ 0.6 μs). In the last 0.3 μs of the simulation, ICL3, which was observed to be the most mobile region of the receptor during the whole simulation, preserved its close state. Principal component analysis of 1 μs long MD trajectory showed that the first principal mode, which explains 69% of the overall motion governs the transition from the initial inactive state to the “very inactive” state.

At this point it may be argued that our simulation conditions, such as the absence of any ligand at the binding site and/or intracellular proteins that may interact with ICL3, depict a non-physiological environment. Even though, the receptor function is clearly linked with its interacting partners, our aim was to elucidate the *intrinsic* conformational dynamics of the intact receptor. Based on the widely accepted population shift mechanism [35-37], we tried to uncover the pre-existing conformational states of the apo receptor, which may be shifted and/or modified by the presence of binding partner(s). In fact, our ANM analysis using different conformers/models of ICL3 strengthened our MD results on receptor dynamics. Either the first or second collective mode in ANM was found to be coupled with the specific motion of ICL3, independent of the model used. In contrast, none of these conformational transitions, nor any allosteric coupling between intra- and extracellular parts, were observed in the clipped model simulation lacking ICL3 region. Thus, we stress that the presence of ICL3 provides a more realistic constriction than those of clipped and non-clipped (loose ends) models so far used in β_2_AR simulations.

Future works will be focusing on the loop model, which will consist of constraining the binding site region to observe the reverse transition (or release) from the “very inactive” state to the inactive/intermediate state and possibly the active state with an opening of the G-protein binding site.

## Methods

### Preparation of the receptor models

The X-ray crystallographic structure of human β_2_AR in complex with T4 lysozyme (T4L) (PDB:2RH1) at 2.40 Å resolution [3] was used as the initial conformation. After removal of T4L, the missing intracellular loop region (ICL3) between residues 231 and 262 was modeled as an unstructured loop of 32-residues length via MODELLER [38]. Even though ICL3 possibly exists as an unstructured loop [33], our modeled loop in Figure 2a interestingly resembles a conformer generated from a fully extended chain by Dror *et al.*[18]. Moreover, it is expected that ICL3 can sample various conformations during our 1 μs long run.

In the second model, Leu230 and Lys263, which are the two ends of the missing region, were covalently attached to each other via a peptide bond to form the “clipped” model (Figure 2b), which has been commonly used in simulation studies.

### Preparation of the environment with the receptor

Each model was later embedded into a palmitoyl­oleoyl-phosphatidylcholine (POPC) membrane bilayer along the z-axis using VMD’s Membrane Plug-in Tool, v1.1 [39]. The receptor was positioned with an oblique angle of 8**°** between its main principal component along the membrane and the z-axis [40]. A total of fifteen internal water molecules detected experimentally in the crystal structure were retained because they make hydrogen bonds with the most conserved residues of the receptor and thus possibly contribute to its structural stability. Using VMD’s solvate module, the protein-lipid system was solvated in both intracellular and extracellular sides with a thickness of 15 Å and 13 Å for the “loop” and “clipped” model, respectively. Finally, the protein-lipid-water system was ionized with Na^+^ and Cl^-^ ions to make the total charge of the system to be equal to zero, which is necessary for Particle-Mesh Ewald summation method used in electrostatic energy calculations. The resulting periodic box dimensions were (86×86×100) and (77×69×90) in Angstrom for the “loop” and “clipped” models, respectively.

### Molecular dynamics simulations

Using the suggested procedure for membrane protein system preparation [41], both models were subjected to three preparation stages. The system consisted of three components of different types, each having a different response time to outside forces. Thus, to reach the equilibrium fast, it was practical to keep some components fixed, while other components were free to move. The first equilibrium stage consisted of melting the lipid tails where only the lipids were free to relax while the protein and waters were held fixed. At the end of the simulation, the unrealistically aligned lipid molecules, transformed into a more disordered, liquid-like structure. In the second preparation step, the protein’s motion was constrained while lipid and water molecules were free to move. Finally in the third stage, the protein was released, and all components were allowed to relax. In every preparation stage, the system was subjected to 1000 steps of energy minimization followed by 0.5 ns MD simulation. At the end of the third stage, the area per lipid was stabilized at 0.635 nm^2^/lipid in agreement with the experimentally measured value of 0.65 nm^2^/lipid [42]. Also, the surface area of the membrane in -*xy* directions decreased due to close packing of lipid molecules with the protein.

Each model was later subjected to 1 μs MD simulation with NAMD v2.7 software tool [41]. CHARMM22 [43,44] and CHARMM27 [45,46] forcefields were used to describe the interaction potential of the protein and the lipid respectively, and waters were treated explicitly using TIP3P model [47]. The system was composed of a total of 68,001 and 42,701 atoms for the “loop” and “clipped” model, respectively. MD simulation was performed at constant NPT at 310 K using Langevin dynamics for all non-hydrogen atoms, with a Langevin damping coefficient of 5 ps^-1^. The system was kept at a constant pressure of 1 atm by using a Nose–Hoover Langevin piston [48] with a period of 100 fs and damping timescale of 50 ps. Long-range electrostatic interactions were treated by particle mesh Ewald (PME) method, with a grid point density of over 1 Å. A cutoff of 12 Å was used for van der Waals and short-range electrostatics interactions with a switching function. Time step was set to 2 fs by using SHAKE algorithm for bonds involving hydrogens [49] and the data was recorded at every 200 ps. The number of time steps between each full electrostatics evaluation was set to 2. Short-range non-bonded interactions were calculated at every time step.

For the “loop model”, three additional 100 ns long MD simulations starting with different initial velocities were performed alongside 1 μs MD simulation. The aim was to possibly explore different conformational subspaces than those visited during the long trajectory of the loop model.

### Docking calculations

Docking was performed using the software tool AutoDock v4.0 [50]. The docking site was selected based on the location of the partial inverse agonist carazolol in the complex structure (PDB:2RH1). Two distinct snapshots taken from MD trajectory were used as target structures. Lamarckian genetic algorithm was used to explore the conformational space. A total of 100 runs were performed for each structure with each run consisting of 1.0×10^6^ and 1.5×10^6^ energy evaluations for epinephrine and ICI ligands, respectively. Grid box constructed with a spacing of 0.375 Å had dimensions of 24 Å × 24 Å × 24 Å for all dockings. For each docking experiment, the pose with the highest score (lowest binding energy of AutoDock) was used as the most probable solution for that complex.

### Elastic network analysis

The collective/global modes of the protein were extracted via the anisotropic network model (ANM) [51,52], which describes the protein as a coarse-grained elastic network of harmonic springs based on a minimum-energy folded conformation. The network is formed simply, by connecting the close-neighboring alpha-carbon atom (called nodes) pairs in the folded structure. The slow or the low-frequency modes extracted from normal mode analysis of the elastic network are known to successfully describe the functional conformational changes.

In our current work, the receptor’s loop model was embedded into a coarse-grained membrane environment according to the methodology provided in Lezon *et al.*[53] The membrane consisting of spheres arranged in an FCC lattice had a diameter of 80 Å and a thickness of 33 Å. The cutoff value for pairwise interactions between nodes was taken as 11 Å. The force constants of harmonic springs were selected as 1.0, 2.0 and 4.0 for protein-protein, protein-membrane and membrane-membrane type of pairwise interactions, respectively. In this model, the membrane environment serves as a constriction and thereby inhibits the unrealistically large fluctuations of the transmembrane helices that would be observed if ANM were applied to the protein alone [53].

## Abbreviations

GPCRs: G protein coupled receptors; β2AR: β_2_-adrenergic receptor; ICL: Intracellular loop; T4L: T4-lysozyme; MD: Molecular dynamics; POPC: Palmitoyl­oleoyl-phosphatidylcholine; TMEMB: Transmembrane region; RMSD: Root mean square deviation; RMSF: Root mean square fluctuation; ECL: Extracellular loop; TM: Transmembrane helix; PCA: Principal component analysis; ANM: Anisotropic network model.

## Competing interests

The authors declare that they have no competing interests.

## Author’s contributions

OO have carried out the molecular dynamics simulations of loop and clipped models. AU performed the anisotropic network analysis. EDA performed the docking calculations. All authors have participated in the analysis and interpretation of MD trajectory, and writing of the manuscript. All authors read and approved the final manuscript.

## Supplementary Material

Additional file 1: Figure S1 Secondary structure profile in the loop model. Small helical formations are observed in ICL3. (Color scale: 0 = turn, 1 = coil, 2 = isolated bridge, 3 = beta sheet, 4 = alpha helix, 5 = 3–10 helices, 6 = Pi helix). Click here for file

Additional file 2: Figure S2 Correlation between RMSD values of ICL2 and lower TM6. (a) loop, and (b) clipped model. (c) Snapshots showing ICL2 in the loop model, shown from intracellular side. Click here for file

Additional file 3: Figure S3 Correlation between RMSD value of ECL2 and Ser207(Oγ)-Asp113(Cγ) distance. (a) loop, and (b) clipped model. Click here for file

Additional file 4: Figure S4 Ionic lock profiles. (a) loop and (b) clipped model. The ionic lock is between guanidinium nitrogen of Arg131 and carboxylate oxygen of Glu268. Profiles of *Χ* angle of Glu268 for the (c) loop and (d) clipped models, respectively. Click here for file

Additional file 5: Figure S5 Essential dynamics of the clipped model. (a) RMSD profile for the lower part of TM6 in the clipped model’s original trajectory (blue) and after the projection onto the first (red) and the cumulative five (green) principal modes. (b) Original and reconstructed profiles for the distance between Ser207(Cα) and Asp113(Cα). (c) The correlation plot between RMSD of TM6 and Ser207-Asp113 distance. (d) Projection of the clipped model’s trajectory onto the first principal mode, shown as harmonic motion. Click here for file

Additional file 6: Figure S6 The loop overlap matrices between the first five modes of PCA (of 1 μs MD run) and the first 20 slowest modes of ANM performed on (A) average structure of 1 μs MD run, (B) alternative loop model from MODELLER, (C) initial and (D) final frames from 1 μs MD run. The loop overlap is calculated as the correlation cosine between the eigenvectors for the specific region including ICL3 and intracellular part of TM6. Click here for file

Additional file 7: Figure S7 Clustering profile of all trajectories based on the binding site region. Click here for file

Additional file 8: Figure S8 Representative snapshots which are closest to the average structure (centroid) of each three clusters in Figure 8B for the loop model. (A) active crystal structure (PDB id: 3SN6), snapshots taken at (B) 52.8 ns (cluster #3), (C) 524 ns (cluster #1) and (D) 806 ns (cluster #2). The gamma subunit of G protein is partly shown in red. All ICL3 regions are colored in a darker tone. Click here for file

Additional file 9: Figure S9 Ligand-receptor interactions. (A, B) for the best poses of epinephrine, and (C, D) for the best poses of ICI in open and closed forms, shown in Figure 9. Click here for file

## References

[B1] Palczewski K Kumasaka T Hori T Behnke CA Motoshima H Fox BA Trong IL Teller DC Okada T Stenkamp RE Yamamoto M Miyano M Crystal structure of rhodopsin: a G protein-coupled receptor Science 2000 289 739 745 10.1126/science.289.5480.739 10926528

[B2] Teller DC Okada T Behnke CA Palczewski K Stenkamp RE Advances in determination of a high-resolution three-dimensional structure of rhodopsin, a model of G-protein-coupled receptors (GPCRs) Biochemistry 2001 40 7761 7772 10.1021/bi0155091 11425302 PMC1698954

[B3] Cherezov V Rosenbaum DM Hanson MA Rasmussen SG Thian FS Kobilka TS Choi HJ Kuhn P Weis WI Kobilka BK Steven RC High-resolution crystal structure of an engineered human beta2-adrenergic G protein-coupled receptor Science 2007 318 1258 1265 10.1126/science.1150577 17962520 PMC2583103

[B4] Rasmussen SG Choi HJ Rosenbaum DM Kobilka TS Thian FS Edwards PC Burghammer M Ratnala VR Sanishvili R Fischetti RF Schertler GF Weis WI Kobilka BK Crystal structure of the human beta2 adrenergic G-protein-coupled receptor Nature 2007 450 383 387 10.1038/nature06325 17952055

[B5] Hanson MA Cherezov V Griffith MT Roth CB Jaakola VP Chien EY Velasquez J Kuhn P Stevens RC A specific cholesterol binding site is established by the 2.8 A structure of the human beta2-adrenergic receptor Structure 2008 16 897 905 10.1016/j.str.2008.05.001 18547522 PMC2601552

[B6] Warne T Serrano-Vega MJ Baker JG Moukhametzianov R Edwards PC Henderson R Leslie AG Tate CG Schertler GF Structure of a beta1-adrenergic G-protein-coupled receptor Nature 2008 454 486 491 10.1038/nature07101 18594507 PMC2923055

[B7] Bokoch MP Zou Y Rasmussen SG Liu CW Nygaard R Rosenbaum DM Fung JJ Choi H Thian FS Kobilka TS Puglisi JD Weis WI Pardo L Prosser RS Mueller L Kobilka BK Ligand-specific regulation of the extracellular surface of a G-protein-coupled receptor Nature 2010 463 108 112 10.1038/nature08650 20054398 PMC2805469

[B8] Wacker D Fenalti G Brown MA Katritch V Abagyan R Cherezov V Stevens RC Conserved binding mode of human beta2 adrenergic receptor inverse agonists and antagonist revealed by X-ray crystallography J Am Chem Soc 2010 132 11443 11445 10.1021/ja105108q 20669948 PMC2923663

[B9] Rosenbaum DM Zhang C Lyons JA Holl R Aragao D Arlow DH Rasmussen SG Choi HJ Devree BT Sunahara RK Chae PS Gellman SH Dror RO Shaw DE Weis WI Caffrey M Gmeiner P Kobilka BK Structure and function of an irreversible agonist-beta(2) adrenoceptor complex Nature 2011 469 236 240 10.1038/nature09665 21228876 PMC3074335

[B10] Rasmussen SG DeVree BT Zou Y Kruse AC Chung KY Kobilka TS Thian FS Chae PS Pardon E Calinski D Mathiesen JM Shah ST Lyons JA Caffrey M Gellman SH Steyaert J Skiniotis G Weis WI Sunahara RK Kobilka BK Crystal structure of the beta2 adrenergic receptor-Gs protein complex Nature 2011 477 549 555 10.1038/nature10361 21772288 PMC3184188

[B11] Rasmussen SG Choi HJ Fung JJ Pardon E Casarosa P Chae PS Devree BT Rosenbaum DM Thian FS Kobilka TS Schnapp A Konetzki I Sunahara RK Gellman SH Pautsch A Steyaert J Weis WI Kobilka BK Structure of a nanobody-stabilized active state of the beta(2) adrenoceptor Nature 2011 469 175 180 10.1038/nature09648 21228869 PMC3058308

[B12] Bahar I Chennubhotla C Tobi D Intrinsic dynamics of enzymes in the unbound state and relation to allosteric regulation Curr Opin Struct Biol 2007 17 633 640 10.1016/j.sbi.2007.09.011 18024008 PMC2197162

[B13] De Lean A Stadel JM Lefkowitz R A ternary complex model explains the agonist-specific binding properties of the adenylate cyclase-coupled beta-adrenergic receptor J Biol Chem 1980 255 7108 7117 6248546

[B14] Ghanouni P Gryczynski Z Steenhuis JJ Lee TW Farrens DL Lakowicz JR Kobilka BK Functionally different agonists induce distinct conformations in the G protein coupling domain of the beta 2 adrenergic receptor J Biol Chem 2001 276 24433 24436 10.1074/jbc.C100162200 11320077

[B15] Swaminath G Xiang Y Lee TW Steenhuis J Parnot C Kobilka BK Sequential binding of agonists to the beta2 adrenoceptor. Kinetic evidence for intermediate conformational states J Biol Chem 2004 279 686 691 14559905 10.1074/jbc.M310888200

[B16] Swaminath G Deupi X Lee TW Zhu W Thian FS Kobilka TS Kobilka B Probing the beta2 adrenoceptor binding site with catechol reveals differences in binding and activation by agonists and partial agonists J Biol Chem 2005 280 22165 22171 10.1074/jbc.M502352200 15817484

[B17] Dror RO Arlow DH Borhani DW Jensen MO Piana S Shaw DE Identification of two distinct inactive conformations of the beta2-adrenergic receptor reconciles structural and biochemical observations Proc Natl Acad Sci USA 2009 106 4689 4694 10.1073/pnas.0811065106 19258456 PMC2650503

[B18] Dror RO Arlow DH Maragakis P Mildorf TJ Pan AC Xu H Borhani DW Shaw DE Activation mechanism of the beta2-adrenergic receptor Proc Natl Acad Sci USA 2011 108 18684 18689 10.1073/pnas.1110499108 22031696 PMC3219117

[B19] Nygaard R Zou Y Dror RO Mildorf TJ Arlow DH Manglik A Pan AC Liu CW Fung JJ Bokoch MP Sun TT Shaw DE Mueller L Prosser RS Kobilka BK The dynamic process of β_2_-adrenergic receptor activation Cell 2013 152 532 542 10.1016/j.cell.2013.01.008 23374348 PMC3586676

[B20] Rosenbaum DM Cherezov V Hanson MA Rasmussen SG Thian FS Kobilka TS Choi HJ Yao XJ Weis WI Stevens RC Kobilka BK GPCR engineering yields high-resolution structural insights into beta2-adrenergic receptor function Science 2007 318 1266 1273 10.1126/science.1150609 17962519

[B21] O’Dowd BF Hnatowich M Regan JW Leader WM Caron MG Lefkowitz RJ Site-directed mutagenesis of the cytoplasmic domains of the human beta 2-adrenergic receptor. Localization of regions involved in G protein-receptor coupling J Biol Chem 1988 263 15985 15992 2846532

[B22] Liggett SB Caron MG Lefkowitz RJ Hnatowich M Coupling of a mutated form of the human beta 2-adrenergic receptor to Gi and Gs. Requirement for multiple cytoplasmic domains in the coupling process J Biol Chem 1991 266 4816 4821 1848226

[B23] Isin B Estiu G Wiest O Oltvai ZN Identifying ligand binding conformations of the β2-adrenergic receptor by using its agonists as computational probes PLoS One 2012 7 12 e50186 10.1371/journal.pone.0050186 23300522 PMC3534076

[B24] Katritch V Reynolds KA Cherezov V Hanson MA Roth CB Yeager M Abagyan R Analysis of full and partial agonists binding to beta(2)-adrenergic receptor suggests a role of transmembrane helix V in agonist-specific conformational changes J Mol Recognit 2009 22 307 318 10.1002/jmr.949 19353579 PMC2693451

[B25] Simpson LM Wall ID Blaney FE Reynolds CA Modeling GPCR active state conformations: the beta(2)-adrenergic receptor Proteins-Structure Function and Bioinformatics 2011 79 1441 1457 10.1002/prot.22974 21337626

[B26] Amadei A Linssen ABM Berendsen HJC Essential dynamics of proteins Proteins-Structure Function and Genetics 1993 17 412 425 10.1002/prot.340170408 8108382

[B27] Kurkcuoglu Z Bakan A Kocaman D Bahar I Doruker P Coupling between catalytic loop motions and enzyme global dynamics Plos Comput Biol 2012 8 9 e1002705 10.1371/journal.pcbi.1002705 23028297 PMC3459879

[B28] MATLAB 7.10.0.499(R2010a) 2010 Natick, Massachusetts: The MathWorks Inc

[B29] Laskowski RA PDBsum new things Nucleic Acids Res 2009 37 D355 D359 10.1093/nar/gkn860 18996896 PMC2686501

[B30] Swaminath G Lee TW Kobilka B Identification of an allosteric binding site for ZN(2+) on the beta(2) adrenergic receptor J Biol Chem 2003 278 352 356 12409304 10.1074/jbc.M206424200

[B31] MOE 2011.10 2011 1010 Sherbooke St. West, Suite #910, Montreal, QC, Canada, H3A2R7: Chemical Computing Group Inc

[B32] The PyMOL Molecular Graphics System P. 0.99 Schrödinger: LLC

[B33] Schlessinger A Punta M Yachdav G Kajan L Rost B Improved disorder prediction by combination of orthogonal approaches PLoS ONE 2009 4 2 e4433 10.1371/journal.pone.0004433 19209228 PMC2635965

[B34] Linse SS The nobel prize in chemistry 2012 - advanced informationmm 2013 http://www.nobelprize.org/nobel_prizes/chemistry/laureates/2012/advanced-chemistryprize2012.pdf

[B35] Monod J Wyman J Changeux JP On the nature of allosteric transitions: a plausible model J Mol Biol 1965 12 88 118 10.1016/S0022-2836(65)80285-6 14343300

[B36] Weber G Ligand binding and internal equilibriums in protein Biochemistry 1972 11 864 878 10.1021/bi00755a028 5059892

[B37] Ma B Kumar S Nussinov R Folding and binding cascades: shifts in energy landscapes Proc Natl Acad Sci U S A 1999 96 18 9970 9972 10.1073/pnas.96.18.9970 10468538 PMC33715

[B38] Narayanan E John B Mirkovic N Fiser A Ilyin VA Pieper U Stuart AC Marti-Renom MA Madhusudhan MS Yerkovich B Sali A Tools for comparative protein structure modeling and analysis Nucleic Acids Res 2003 31 3375 3380 10.1093/nar/gkg543 12824331 PMC168950

[B39] Humphrey W Dalke A Schulten K VMD: visual molecular dynamics J Mol Graph 1996 14 33 38 10.1016/0263-7855(96)00018-5 8744570

[B40] Lomize MA Lomize AL Pogozheva ID Mosberg HI OPM: orientations of proteins in membranes database Bioinformatics 2006 22 623 625 10.1093/bioinformatics/btk023 16397007

[B41] Phillips JC Braun R Wang W Gumbart J Tajkhorshid E Villa E Chipot C Skeel RD Kalé L Schulten K Scalable molecular dynamics with NAMD J Comput Chem 2005 26 1781 1802 10.1002/jcc.20289 16222654 PMC2486339

[B42] Petrache HI Dodd SW Brown MF Area per lipid and acyl length distributions in fluid phosphatidylcholines determined by (2)H NMR spectroscopy Biophys J 2000 79 3172 3192 10.1016/S0006-3495(00)76551-9 11106622 PMC1301193

[B43] Mackerell AD Bashford D Bellott M Dunbrack RL Evanseck JD Field MJ Fischer S Gao J Guo H Ha S Joseph D Kuchnir L Kuczera K Lau FTK Mattos C Michnick S Ngo T Nguyen DT Prodhom B Roux B Schlenkrich M Smith J Stote R Straub J Watanabe M Wiorkiewicz-Kuczera J Yin D Karplus M Self-consistent parameterization of biomolecules for molecular modeling and condensed phase simulations Faseb Journal 1992 6 A143 A143

[B44] Mackerell AD Bashford D Bellott M Dunbrack RL Evanseck JD Field MJ Fischer S Gao J Guo H Ha S Joseph D Kuchnir L Kuczera K Lau FTK Mattos C Michnick S Ngo T Nguyen DT Prodhom B Roux B Schlenkrich M Smith J Stote R Straub J Watanabe M Wiorkiewicz-Kuczera J Yin D Karplus M All-atom empirical potential for molecular modeling and dynamics studies of proteins J Phys Chem B 1998 102 3586 3616 10.1021/jp973084f 24889800

[B45] Schlenkrich M Brickmann J MacKerell AD Jr Karplus M Merz KMJr, Roux B An empirical potential energy function for phospholipids: criteria for parameter optimization and applications Biological Membranes: A Molecular Perspective from Computation and Experiment 1996 1 Birkhauser, Boston

[B46] Feller SE Yin D Pastor RW MacKerell AD Jr Molecular dynamics simulation of unsaturated lipids at Low hydration: parametrization and comparison with diffraction studies Biophys J 1997 73 2269 2279 10.1016/S0006-3495(97)78259-6 9370424 PMC1181132

[B47] Jorgensen WL Chandrasekhar J Madura JD Impey RW Klein ML Comparison of simple potential functions for simulating liquid water J Chem Phys 1983 79 926 935 10.1063/1.445869

[B48] Feller SE Zhang YH Pastor RW Computer-simulation of liquid/liquid interfaces 2. Surface-tension area dependence of a bilayer and monolayer J Chem Phys 1995 103 10267 10276 10.1063/1.469928

[B49] Ryckaert JP Ciccotti G Berendsen HJC Numerical-integration of cartesian equations of motion of a system with constraints - molecular-dynamics of N-alkanes J Comput Phys 1977 23 327 341 10.1016/0021-9991(77)90098-5

[B50] Morris GM Huey R Lindstrom W Sanner MF Belew RK Goodsell DS Olson AJ AutoDock4 and AutoDockTools4: automated docking with selective receptor flexibility J Comput Chem 2009 30 2785 2791 10.1002/jcc.21256 19399780 PMC2760638

[B51] Doruker P Atilgan AR Bahar I Dynamics of proteins predicted by molecular dynamics simulations and analytical approaches: application to alpha-amylase inhibitor Proteins 2000 40 512 524 10.1002/1097-0134(20000815)40:3<512::AID-PROT180>3.0.CO;2-M 10861943

[B52] Atilgan AR Durell SR Jernigan RL Demirel MC Keskin O Bahar I Anisotropy of fluctuation dynamics of proteins with an elastic network model Biophys J 2001 80 505 515 10.1016/S0006-3495(01)76033-X 11159421 PMC1301252

[B53] Lezon TR Bahar I Constraints imposed by the membrane selectively guide the alternating access dynamics of the glutamate transporter GltPh Biophys J 2012 102 1331 1340 10.1016/j.bpj.2012.02.028 22455916 PMC3309413

