# Supplementary figures and images for: Effect of intracellular loop 3 on intrinsic dynamics of human β2-adrenergic receptor
Source: BMC Struct Biol. 2013 Nov 9;13:29. doi: 10.1186/1472-6807-13-29 (PMC3834532; doi:10.1186/1472-6807-13-29)

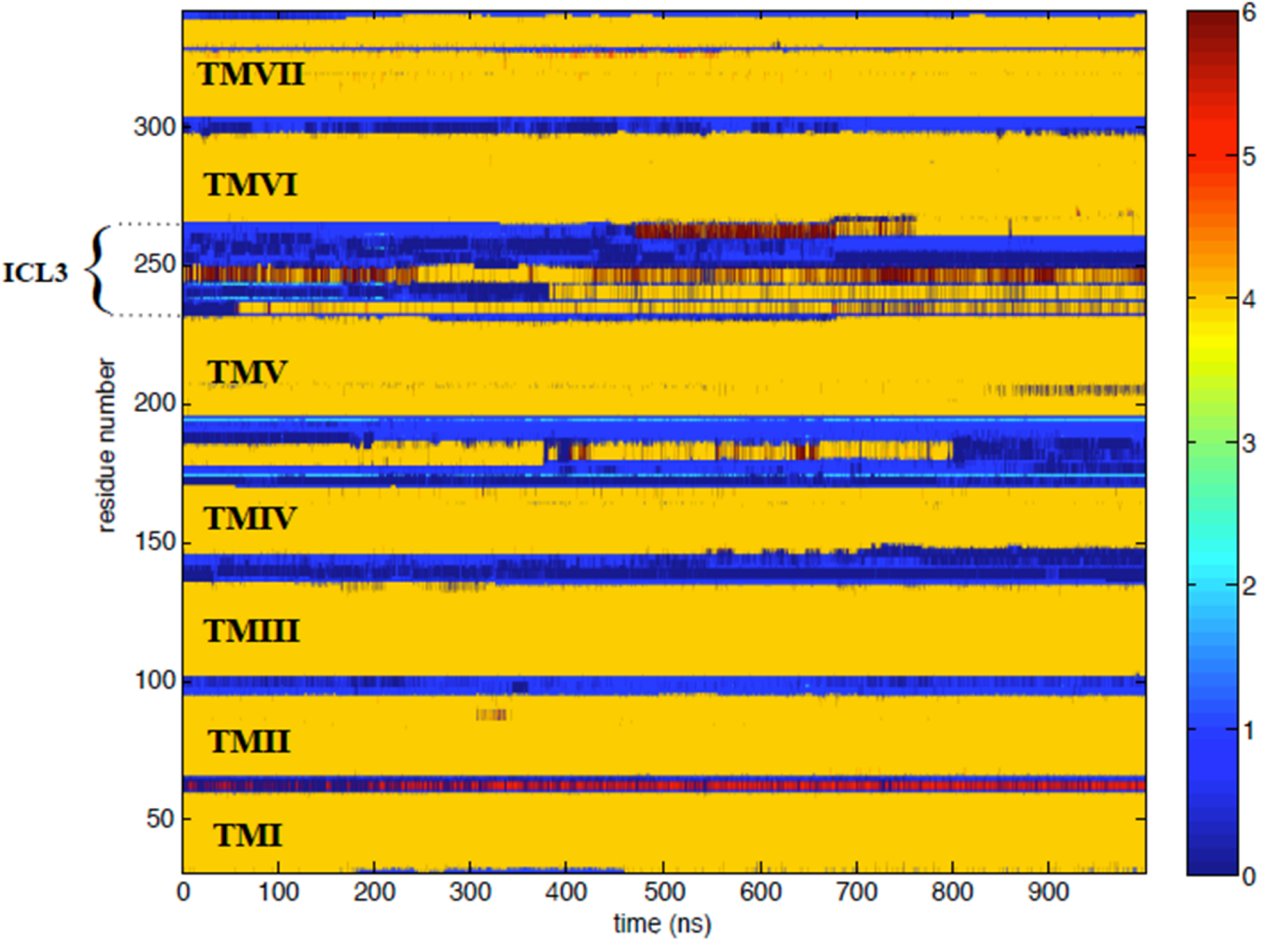

Supplement: Additional file 1: Figure S1 — Secondary structure profile in the loop model. Small helical formations are observed in ICL3. (Color scale: 0 = turn, 1 = coil, 2 = isolated bridge, 3 = beta sheet, 4 = alpha helix, 5 = 3–10 helices, 6 = Pi helix). [file 1472-6807-13-29-S1.pdf]

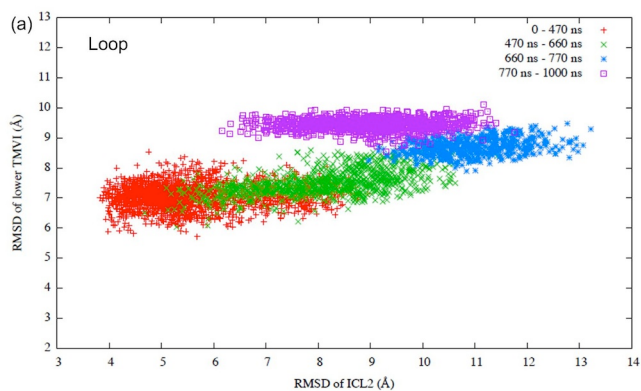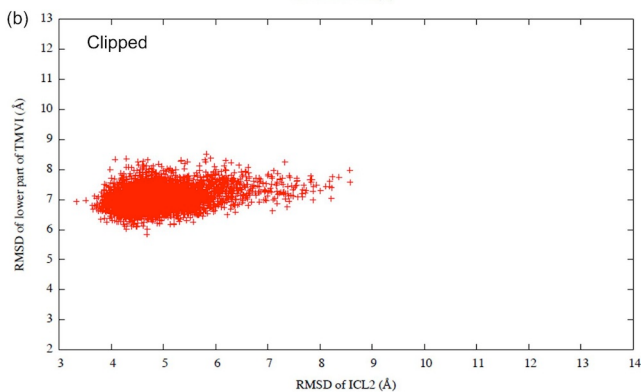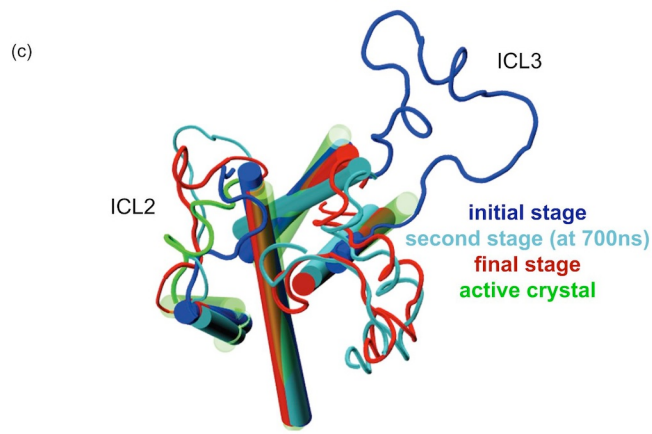

Supplement: Additional file 2: Figure S2 — Correlation between RMSD values of ICL2 and lower TM6. (a) loop, and (b) clipped model. (c) Snapshots showing ICL2 in the loop model, shown from intracellular side. [file 1472-6807-13-29-S2.pdf]

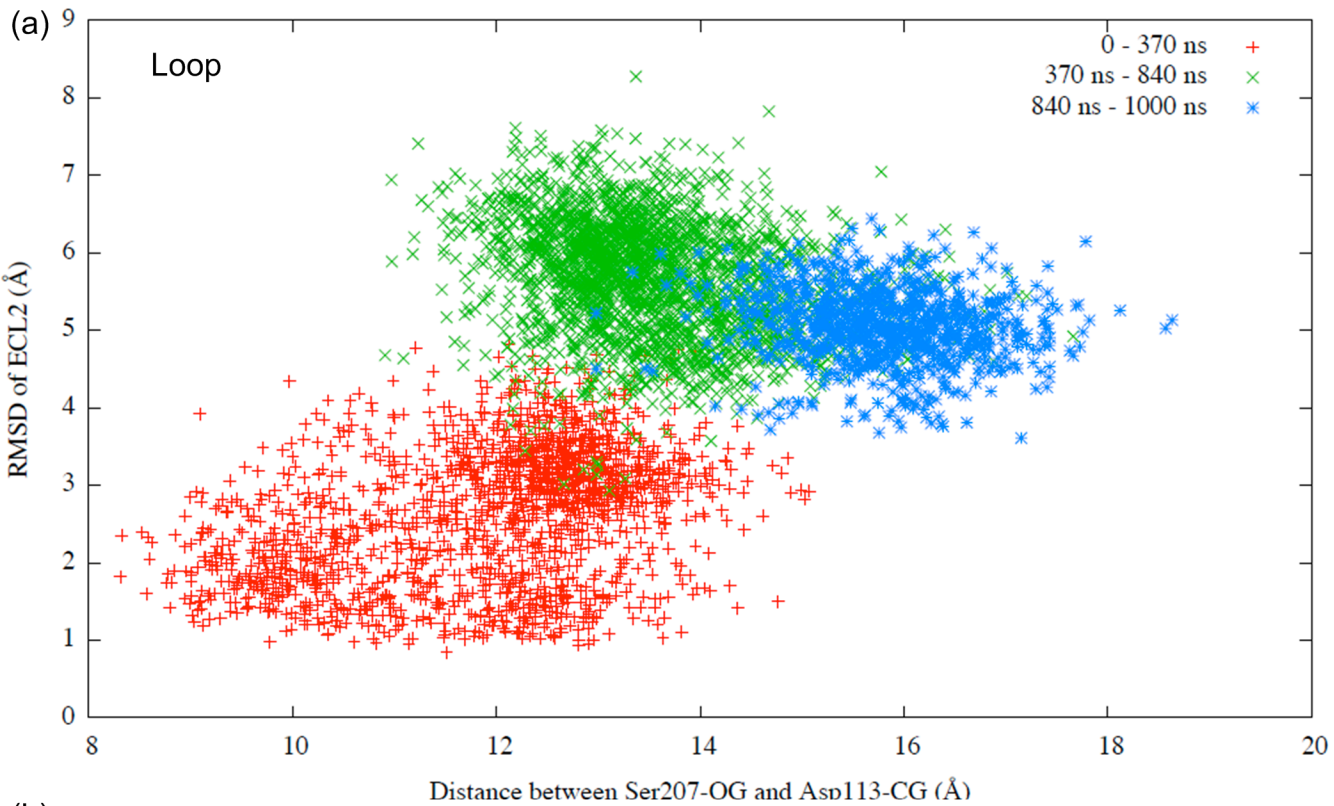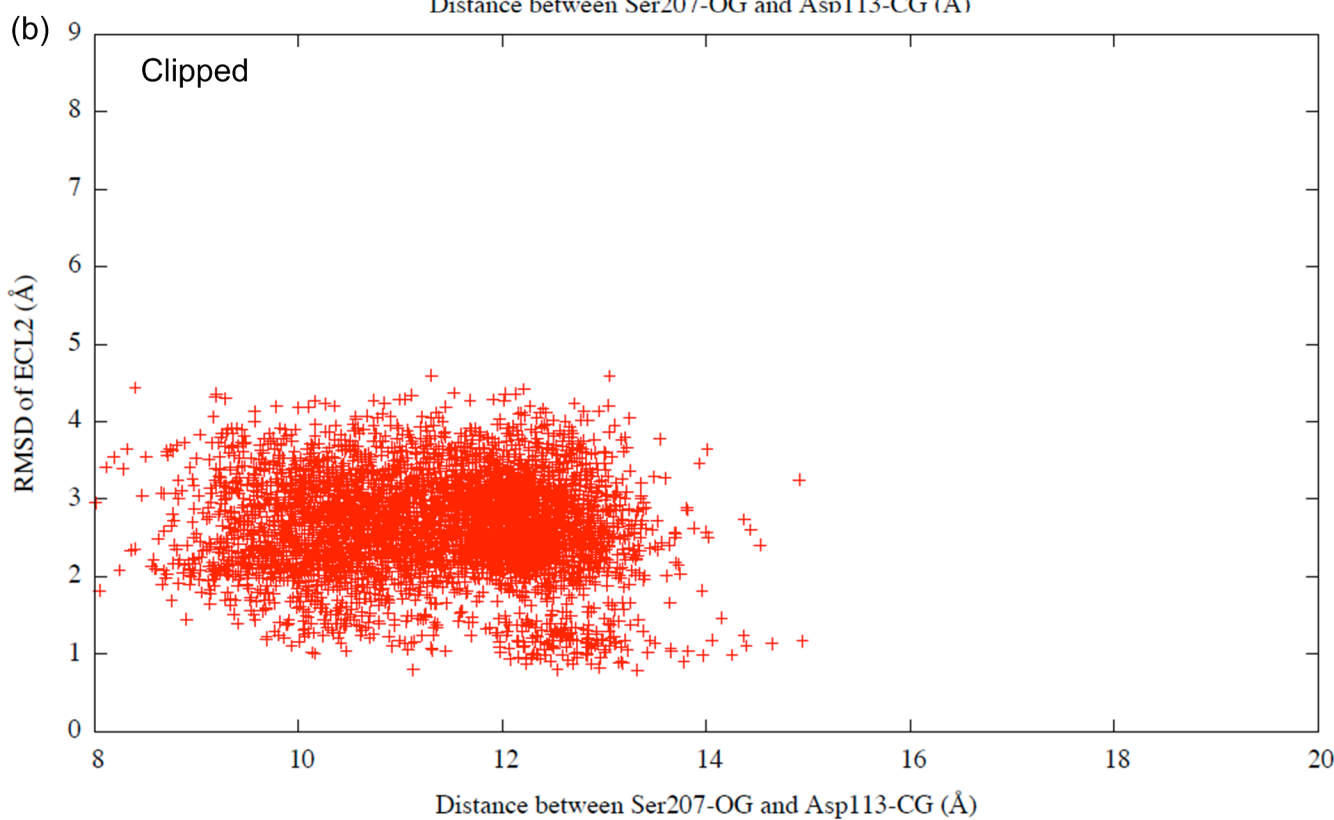

Supplement: Additional file 3: Figure S3 — Correlation between RMSD value of ECL2 and Ser207(Oγ)-Asp113(Cγ) distance. (a) loop, and (b) clipped model. [file 1472-6807-13-29-S3.pdf]

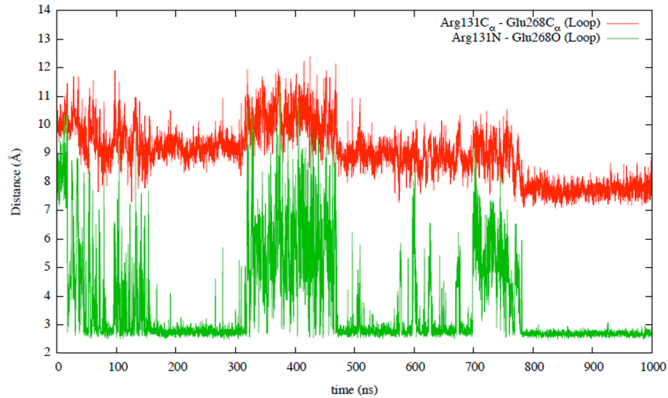

(a)

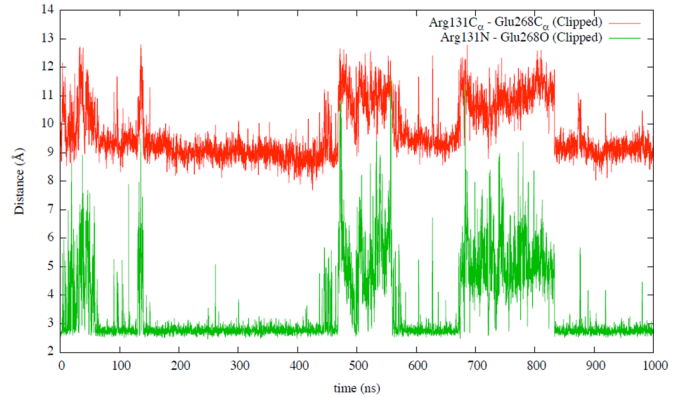

(b)

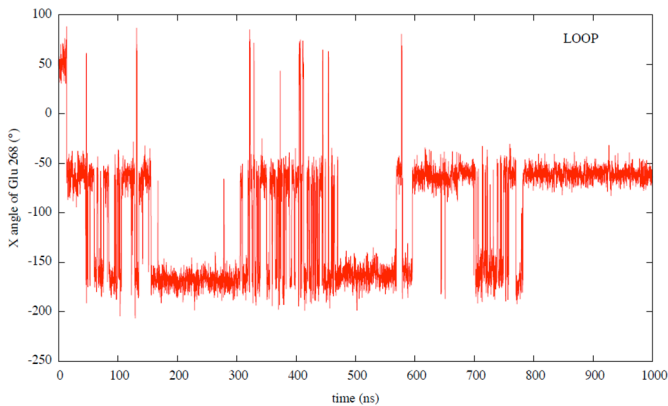

(c)

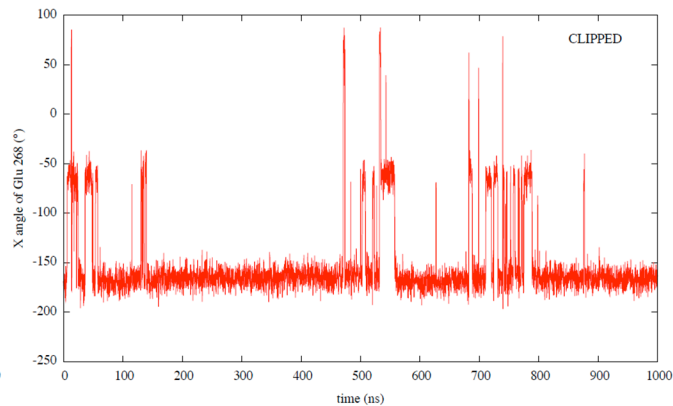

(d)

Supplement: Additional file 4: Figure S4 — Ionic lock profiles. (a) loop and (b) clipped model. The ionic lock is between guanidinium nitrogen of Arg131 and carboxylate oxygen of Glu268. Profiles of Χ angle of Glu268 for the (c) loop and (d) clipped models, respectively. [file 1472-6807-13-29-S4.pdf]

— all modes    — first mode    — cumulative five modes

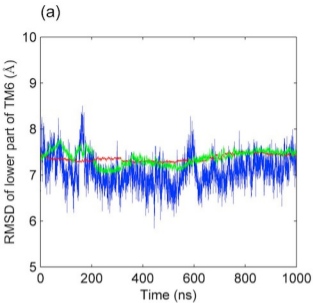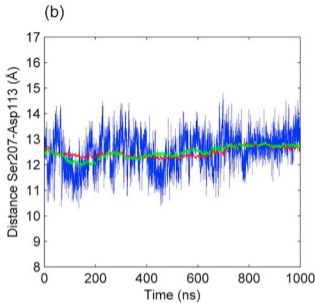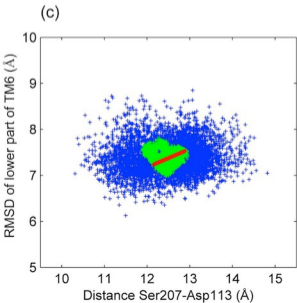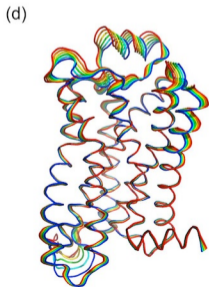

Supplement: Additional file 5: Figure S5 — Essential dynamics of the clipped model. (a) RMSD profile for the lower part of TM6 in the clipped model’s original trajectory (blue) and after the projection onto the first (red) and the cumulative five (green) principal modes. (b) Original and reconstructed profiles for the distance between Ser207(Cα) and Asp113(Cα). (c) The correlation plot between RMSD of TM6 and Ser207-Asp113 distance. (d) Projection of the clipped model’s trajectory onto the first principal mode, shown as harmonic motion. [file 1472-6807-13-29-S5.pdf]

A

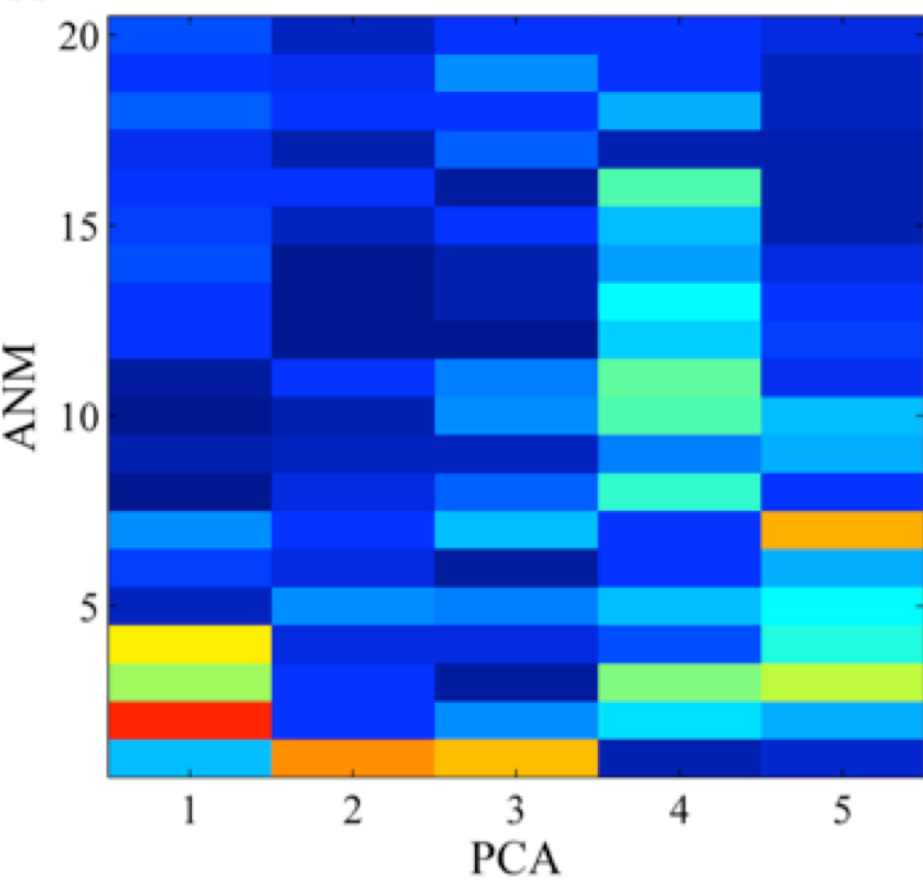

B

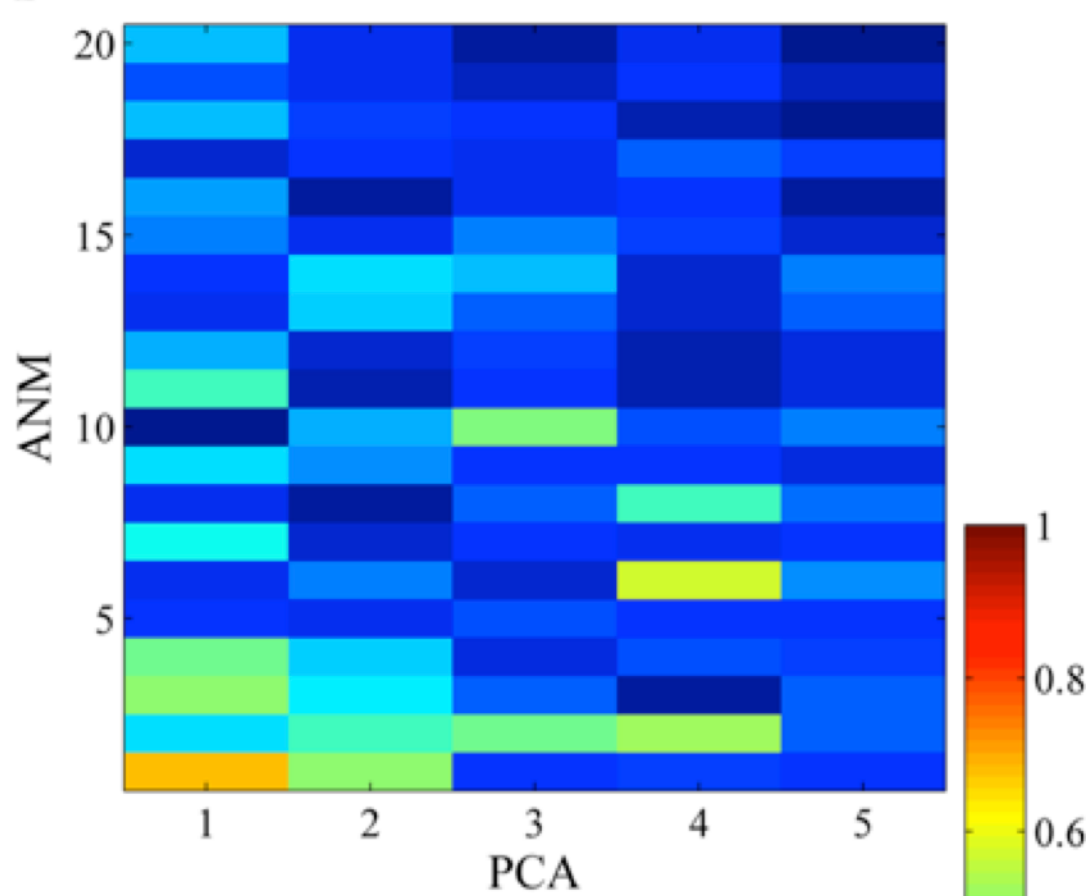

C

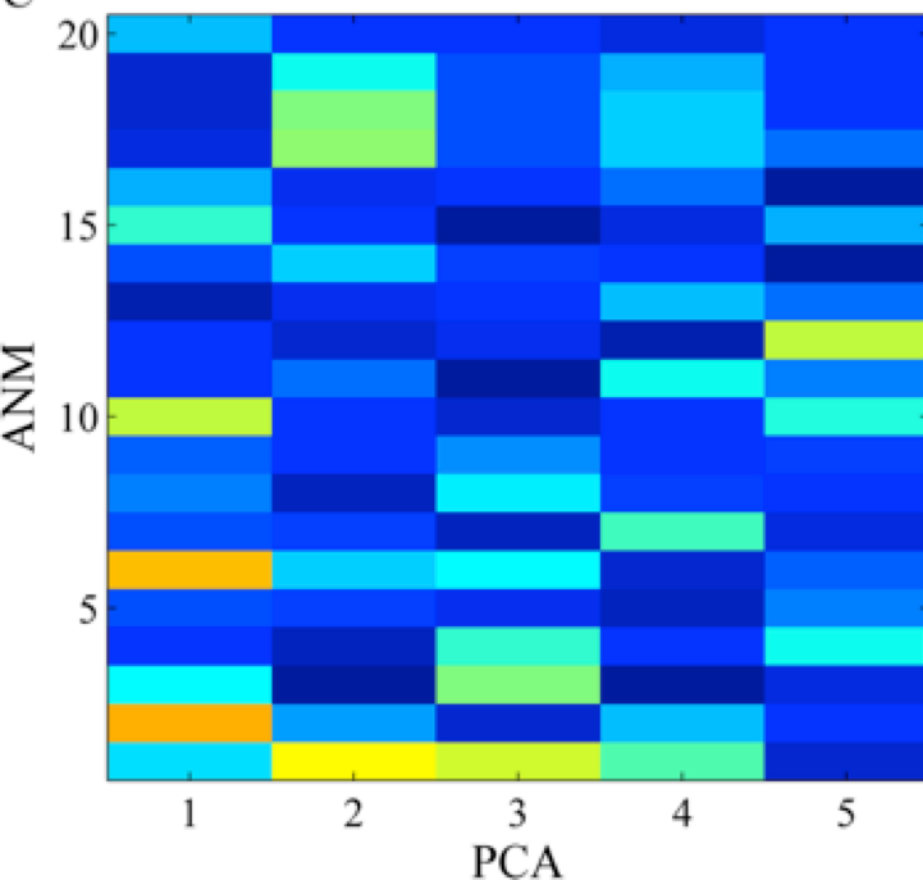

D

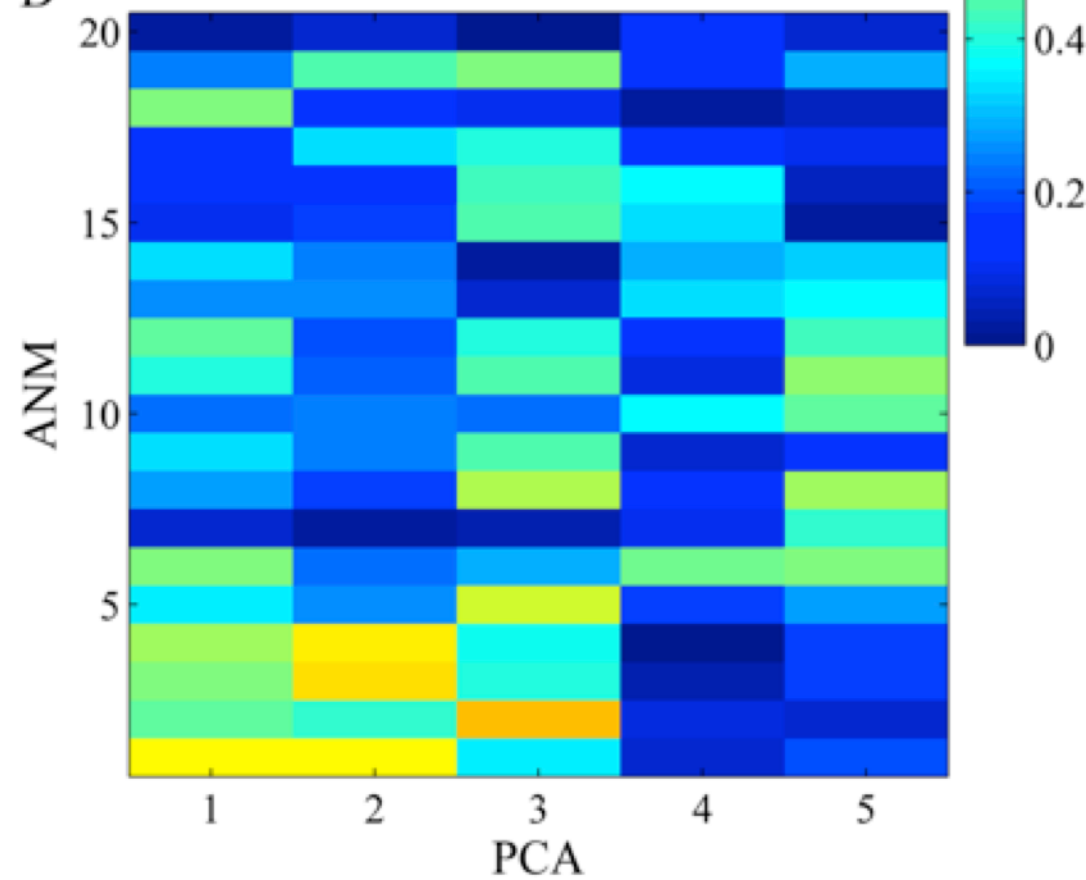

Supplement: Additional file 6: Figure S6 — The loop overlap matrices between the first five modes of PCA (of 1 μs MD run) and the first 20 slowest modes of ANM performed on (A) average structure of 1 μs MD run, (B) alternative loop model from MODELLER, (C) initial and (D) final frames from 1 μs MD run. The loop overlap is calculated as the correlation cosine between the eigenvectors for the specific region including ICL3 and intracellular part of TM6. [file 1472-6807-13-29-S6.pdf]

# BINDING SITE

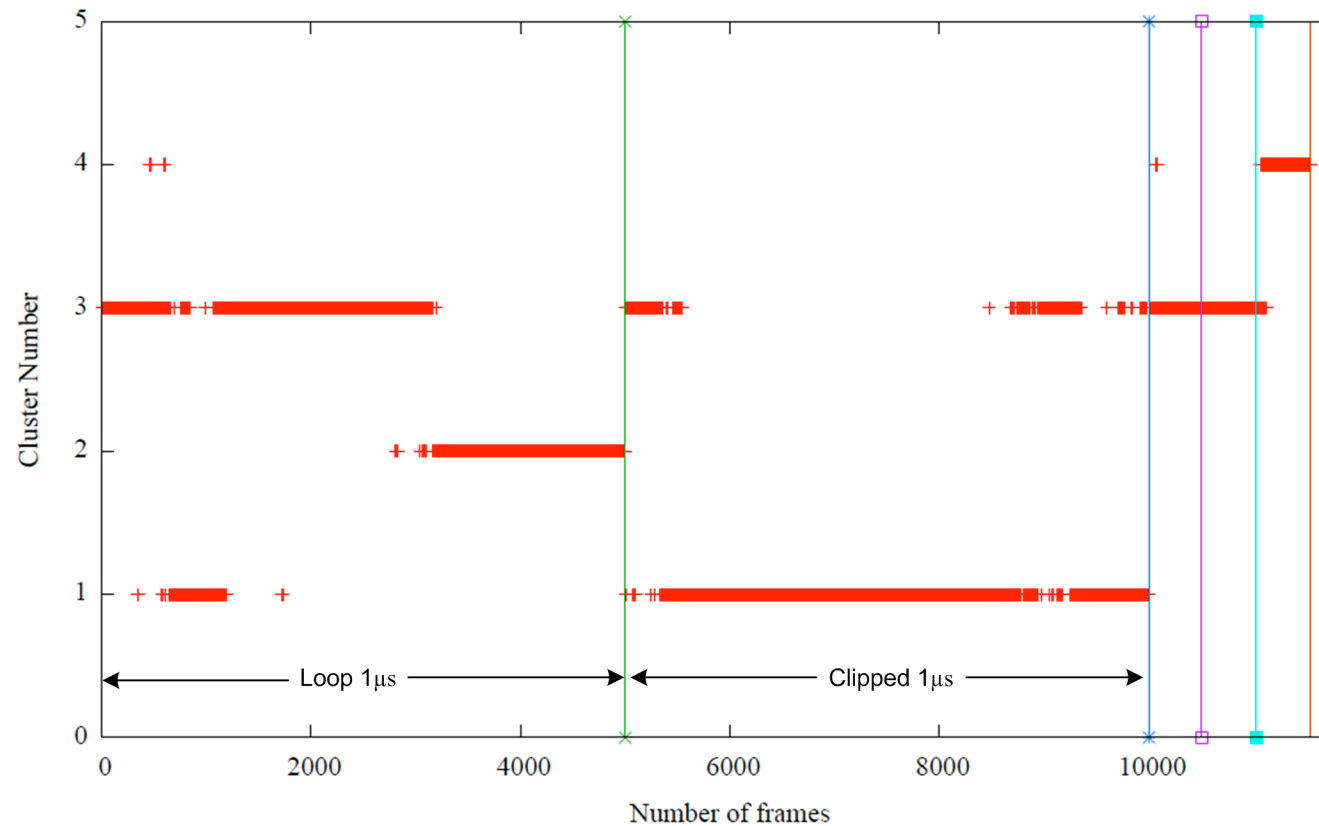

Supplement: Additional file 7: Figure S7 — Clustering profile of all trajectories based on the binding site region. [file 1472-6807-13-29-S7.pdf]

**A**

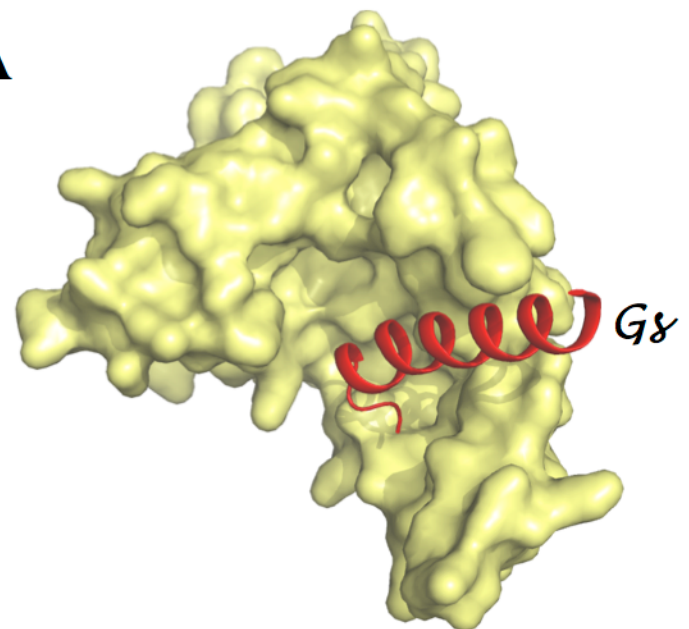

**B**

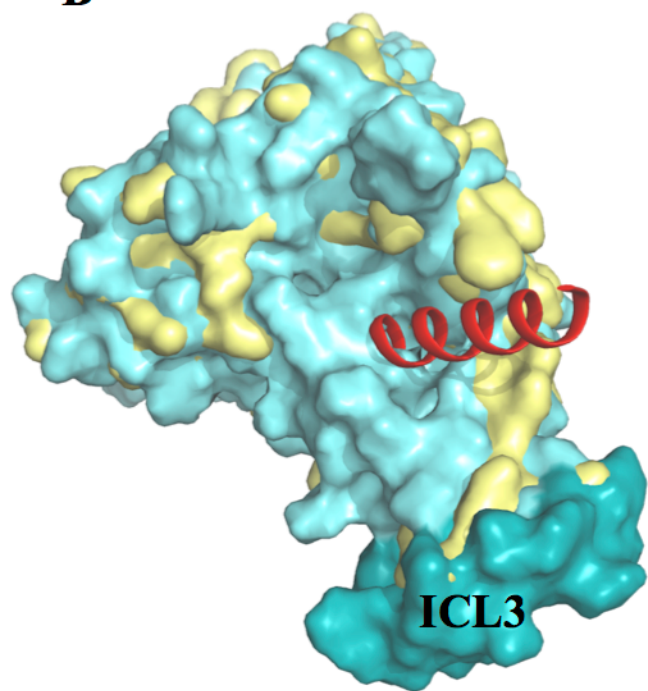

**C**

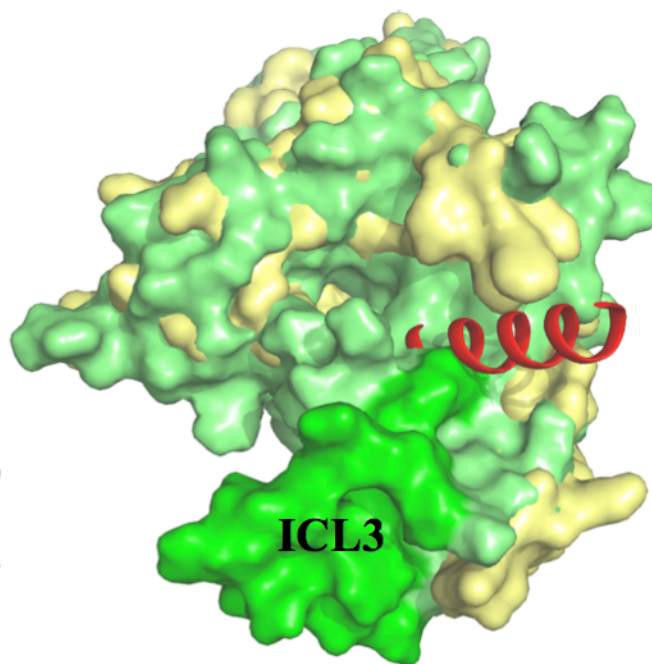

**D**

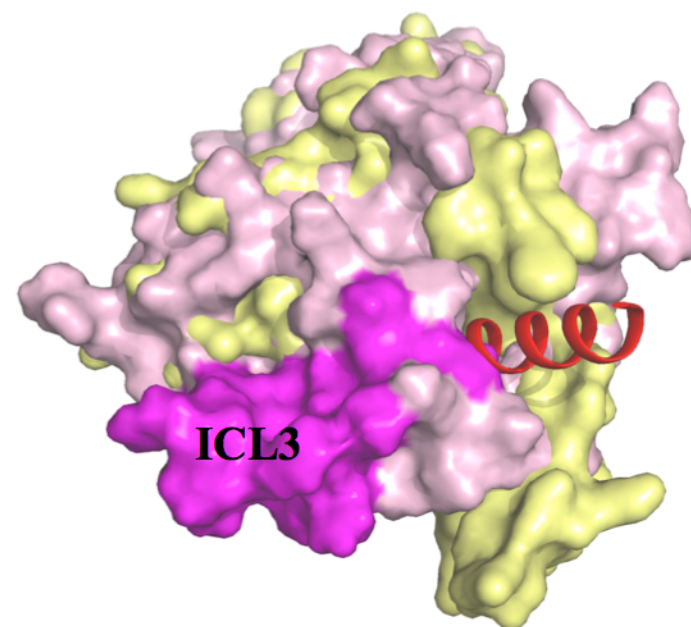

Supplement: Additional file 8: Figure S8 — Representative snapshots which are closest to the average structure (centroid) of each three clusters in Figure 8B for the loop model. (A) active crystal structure (PDB id: 3SN6), snapshots taken at (B) 52.8 ns (cluster #3), (C) 524 ns (cluster #1) and (D) 806 ns (cluster #2). The gamma subunit of G protein is partly shown in red. All ICL3 regions are colored in a darker tone. [file 1472-6807-13-29-S8.pdf]

## Epinephrine interactions

**A**

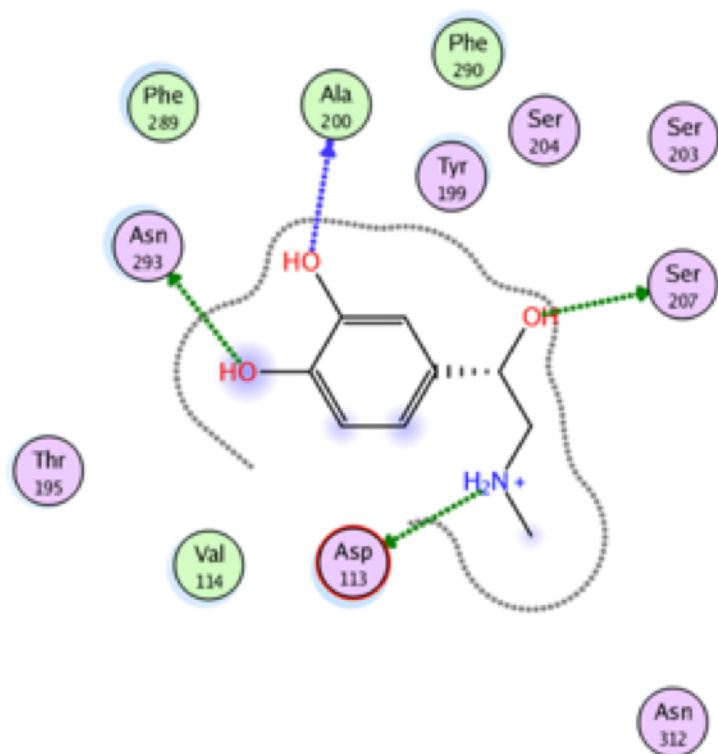

*Closed form*

**B**

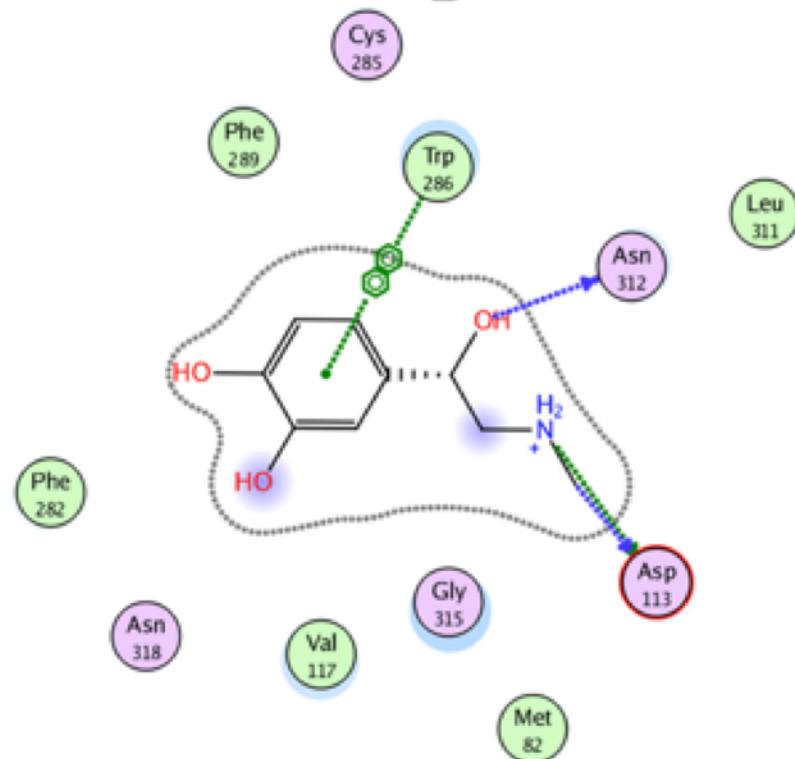

*Open form*

## ICI interactions

**C**

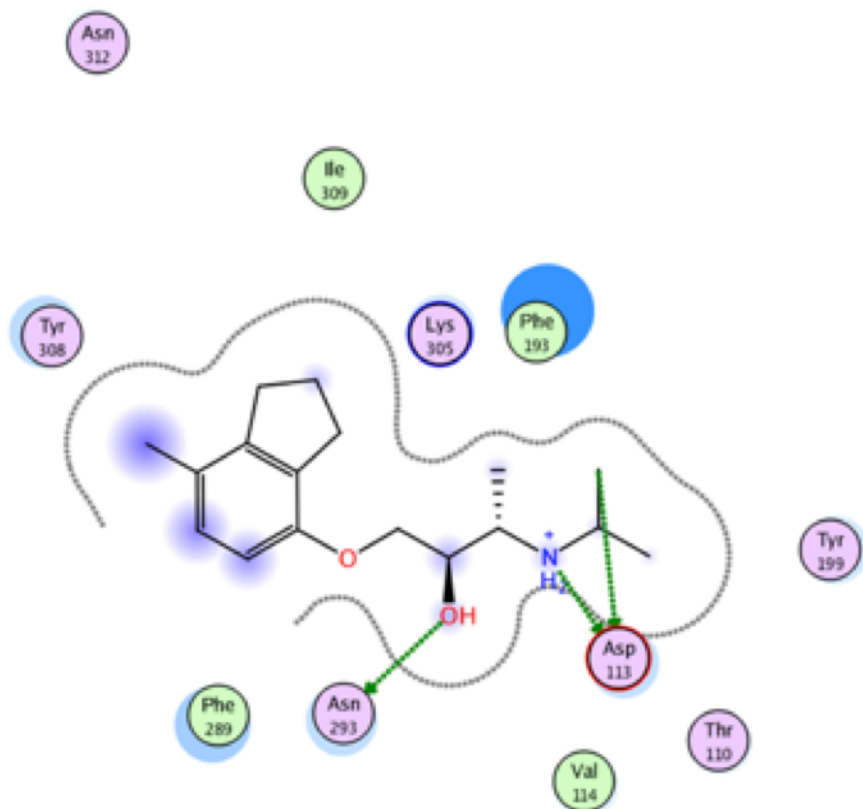

*Closed form*

**D**

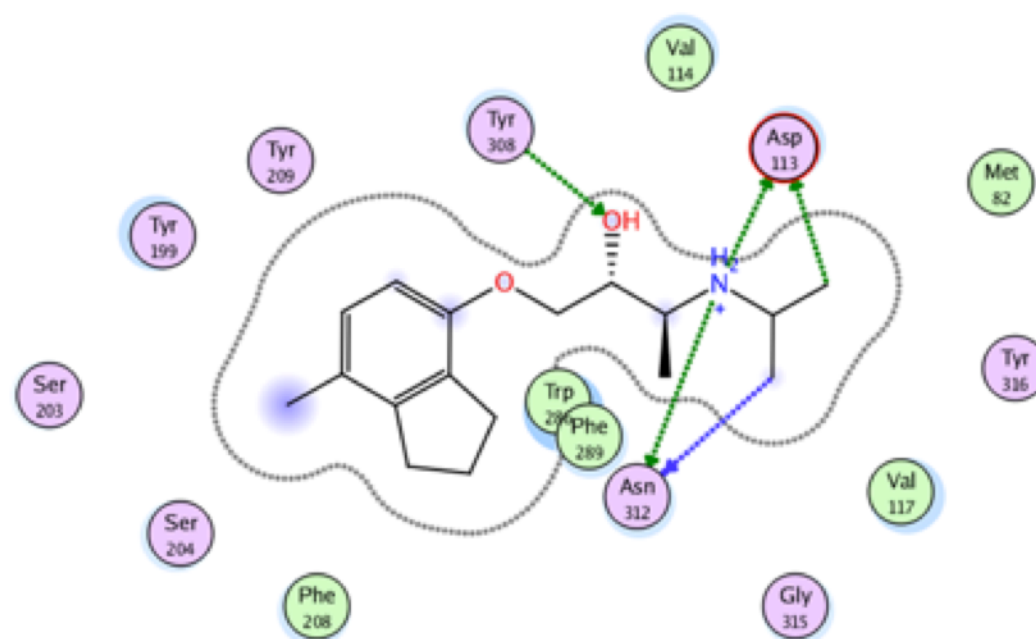

*Open form*

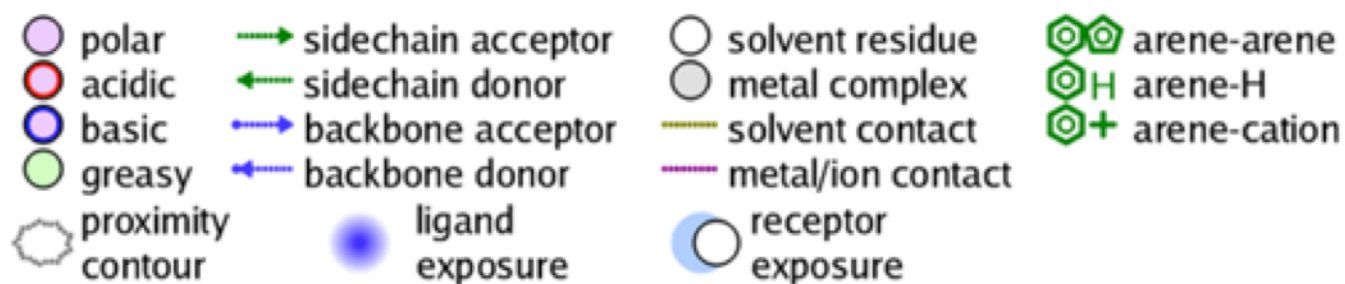

Supplement: Additional file 9: Figure S9 — Ligand-receptor interactions. (A, B) for the best poses of epinephrine, and (C, D) for the best poses of ICI in open and closed forms, shown in Figure 9. [file 1472-6807-13-29-S9.pdf]
